# Oral *Actinomyces* Species Exposure is Associated with Anxiety‐Like Behavior, Mitochondrial Remodeling, and Altered GABAergic Pathways

**DOI:** 10.1002/advs.78144

**Published:** 2026-10-14

**Authors:** Tabinda Salman, Zhenwu Luo, Douglas Johnson, Arshad A. Noorani, Zhuang Wan, Bogdan Bordieanu, Zhi‐wei Ye, Rachel D. Penrod, Hongxu Xian, Sylvia Fitting, Peter W. Kalivas, Wei Jiang

**Affiliations:** ^1^ Department of Pharmacology and Immunology Medical University of South Carolina Charleston South Carolina USA; ^2^ Ralph H. Johnson VA Medical Center Charleston South Carolina USA; ^3^ Department of Neuroscience Medical University of South Carolina Charleston South Carolina USA; ^4^ Versiti Blood Research Institute Medical College of Wisconsin Milwaukee Wisconsin USA; ^5^ Department of Psychology and Neuroscience University of North Carolina at Chapel Hill Chapel Hill North Carolina USA

**Keywords:** anxiety‐like behavior, arginine‐argininosuccinate axis, GABAergic pathways, metabolites, mitochondrial remodeling, oral actinomyces

## Abstract

**Introduction:**

The human oral microbiome is increasingly recognized as a contributor to brain health, yet its mechanisms remain unclear. We previously found an enrichment of oral *Actinomyces* species in chronic cannabis smokers.

**Method:**

Wild‐type C57BL/6 mice were orally inoculated with *Actinomyces* species following antibiotic preconditioning and assessed using behavioral assays, multi‐tissue cytokine profiling, single‐cell RNA sequencing, metabolomics, and biochemical analyses. Selected metabolites were tested in neuronal cells in vitro.

**Results:**

Oral inoculation with cannabis use‐associated *Actinomyces* species, especially *A. meyeri*, in mice was accompanied by anxiety‐like behaviors, alterations in mitochondrial pathways, and reduced GABAergic markers, without evidence of bacterial brain translocation, changes in the canonical proinflammatory cytokines measured in this study, or memory decline. Notably, integrated oral and brain metabolomic analyses prioritized arginine and argininosuccinate as candidate mediators associated with Actinomyces exposure for further investigation. Selected metabolites associated with *Actinomyces* exposure altered mitochondrial function and oxidative stress in neuronal cells in vitro, supporting their potential role in mediating the observed molecular changes.

**Conclusion:**

Together, these results suggest that oral microbiome‐associated metabolites may contribute to behavioral alterations through mechanisms involving mitochondrial remodeling and altered GABAergic pathways, supporting further investigation of the oral‐brain metabolic axis in neuropsychiatric disorders.

## Graphical abstract

Oral exposure to cannabis‐associated Actinomyces species, particularly *A. meyeri*, is associated with anxiety‐like behavior in mice in the absence of detectable bacterial translocation or overt systemic inflammation. These findings support a potential oral‐brain metabolic axis involving candidate metabolites such as arginine and argininosuccinate, accompanied by mitochondrial remodeling and altered GABAergic signaling, providing new insight into potential links between the oral microbiome and neuropsychiatric outcomes.

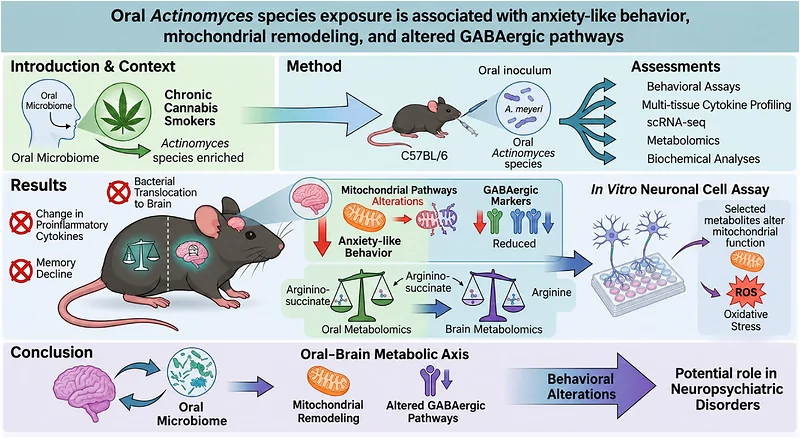

## 1 Introduction

The oral microbiota is reported to be influenced by smoking and linked with various pathophysiological conditions [1, 2]. Drugs of abuse can have a positive or negative impact on the oral microbiome, as well as being associated with a variety of disorders, including cognitive deficiency, anxiety/depression, inflammation, oral disease, or obesity [3]. We previously reported that chronic heavy cannabis smokers showed a disrupted balance of bacteria (specifically enriched *Actinomyces* genus and species) in their mouth [4]. Although the *Actinomyces* genus is considered predominantly commensal in the human oral cavity [5], *Actinomyces* species, such as *A. meyeri*, can act as opportunistic pathogens and are generally reported to be less prominent in the gut due to environmental constraints [6]. Building on these findings, we focused on *A. meyeri* as a candidate oral microbe to investigate whether shifts in oral microbial composition are associated with neurobiological processes relevant to brain function and behavior.

Anxiety disorders are among the most prevalent neuropsychiatric conditions globally, yet the underlying biological mechanisms are often poorly understood [7]. Genetics [8], immune dysregulation [9], and central nervous system (CNS) factors like neurotransmitter imbalances, particularly reduced inhibitory GABAergic signaling, have been implicated in anxiety‐related pathophysiology [10]. In addition to these central mechanisms, emerging evidence highlights the importance of peripheral systems in modulating brain function and behavior. In particular, the gut‐brain axis has been extensively studied as a bidirectional communication pathway involving microbial metabolites, immune signaling, and neural circuits [11]. More recently, the oral‐brain axis has begun to gain attention as a potential contributor to neurobiological regulation, although its mechanistic basis remains less well defined [12].

Therefore, we hypothesized that alterations in the oral microbiota may influence brain function and behavior through oral‐brain communication pathways, potentially involving microbial metabolites. Based on our prior observations of *Actinomyces* enrichment in human oral microbiomes of cannabis smokers [4] and its potential to act as an opportunistic pathogen, we selected *A. meyeri* as a representative species to investigate its neurobiological impact. To test this, we orally inoculated C57BL/6 mice and evaluated neurobehavioral alterations, such as anxiety‐like behavioral symptoms, together with alterations in GABAergic pathways, mitochondrial remodeling, and metabolomic profiles associated with chronic oral Actinomyces exposure.

## 2 Results

### 2.1 Chronic Oral *A. meyeri* Inoculation is Associated with Anxiety‐Like Behavior

Following five days of oral antibiotic treatment to support the target microbiome colonization (Figure 1A), two *Actinomyces* species previously identified as enriched in human oral microbiomes of cannabis smokers (*A. meyeri*, the bacterium of interest, and *A. odontolyticus*), as well as a species reported to be less abundant (*N. elongata*) [4] and a vehicle control were orally inoculated into wild‐type C57BL/6 (B6) mice for six months. Only animals with confirmed colonization (sustained oral enrichment) of the inoculated bacteria were included in the analyses. After confirming successful oral colonization/enrichment with the bacteria of interest (Figure S1), mice underwent behavioral tests (baseline spontaneous locomotor activity, weight, and serum corticosterone levels) after six months of inoculation, prior to the battery of mouse behavioral tests (Figure 1A, Figure S2A). After six months of oral inoculation of *A. meyeri*, mice spent more time in the periphery of the open field test and less time in the absolute center of the apparatus as compared to the vehicle control, while total distance traveled during the open‐field test remained unchanged across groups (Figure 1C). Further analysis using total OFT distance as a covariate (ANCOVA) showed that the overall group effect on absolute‐center time remained significant after adjustment for locomotor activity, whereas the adjusted group effect on periphery time did not reach statistical significance. In addition, *A. meyeri*‐treated mice traveled a greater distance in the peripheral zone, whereas distance traveled in the absolute center showed a similar downward trend. Together, these findings support altered spatial exploration consistent with an anxiety‐like phenotype rather than generalized changes in locomotor activity (Figure 1B,C). The two *Actinomyces* species‐treated mice, when subjected to the elevated plus‐maze test, spent less time in the open arms of the apparatus and more time in the closed arms as compared to the vehicle‐treated control (similar directional pattern), providing additional support for an anxiety‐like phenotype (Figure 1D,E). There was no significant alteration in cognition or depression related behaviors in *Actinomyces* species‐treated mice (Figure S2B–D).

**FIGURE 1:**
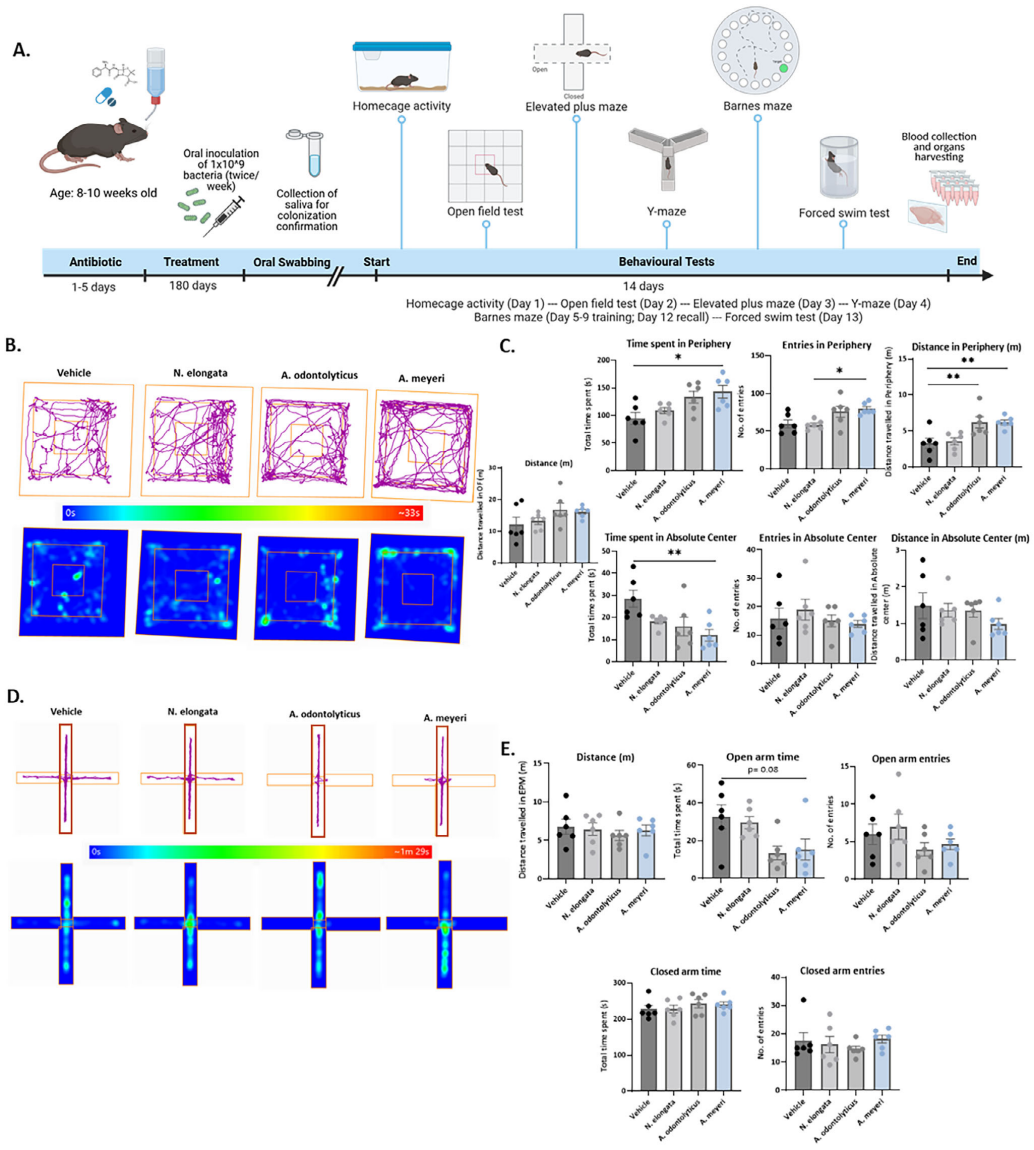
Chronic oral inoculation of *A. meyeri* is associated with an anxiety‐like behavior. (A) Schematic of the mouse study design. Following five days of oral antibiotic pretreatment, mice were chronically inoculated twice weekly for six months with Actinomyces meyeri, Actinomyces odontolyticus, Neisseria elongata, or vehicle control (1 × 10^9 CFU/mL). Oral swabs were collected to confirm sustained oral enrichment. Behavioral testing was performed in order of increasing stress intensity, followed by sacrifice and tissue collection. (B) Representative track plots and heatmaps from the open field test (OFT), illustrating movement patterns and time spent in different zones. (C) Quantification of OFT parameters, including time, entries, and distance traveled in the periphery and absolute center, together with the total distance traveled. A. meyeri–inoculated mice exhibited increased time in the periphery (F*_(3,20)_* = 4.907, p = 0.0103) and reduced time in the center (F*_(3,20)_* = 4.94, p = 0.0100), with increased distance traveled in the peripheral zone (F*_(3,20)_* = 6.993, p = 0.0021), despite no significant difference in total distance traveled. Entries into the peripheral zone were also increased as compared to the *N. elongata* group (F*_(3,20) =_* 4.709, p = 0.0121). To further account for locomotor activity, ANCOVA was performed using total OFT distance traveled as a covariate. The adjusted group effect remained significant for absolute‐center time (F*_(3,19)_* = 4.813, p = 0.0117), whereas the adjusted group effect for periphery time was attenuated (F*_(3,19)_* = 2.865, p = 0.0638). Homogeneity‐of‐regression‐slopes tests were nonsignificant for absolute‐center time (F*_(3,16)_* = 0.7522, p = 0.5370) and periphery time (F*_(3,16)_* = 1.842, p = 0.1802). (D) Representative trajectories and heatmaps from the elevated plus maze (EPM), showing time spent in open versus closed arms. (E) Quantification of EPM parameters, including time, entries, and total distance traveled. For EPM open‐arm time, an overall group effect was detected (F*_(3,20)_* = 4.040, p = 0.0213); however, post hoc comparisons of *A. meyeri* and *A. odontolyticus* with vehicle did not reach statistical significance (p = 0.0848 and p = 0.0509, respectively). No significant group differences were observed in the other EPM parameters. Data are presented as mean ± SEM (*n* = 6 per group). ^*^ *p* < 0.05, ^**^p < 0.01; one‐way ANOVA with Tukey's multiple comparisons test.

### 2.2 Chronic Oral *A. meyeri* Exposure Alters Microglial Markers Without Changes in Proinflammatory Cytokines

We next assessed whether chronic oral *A. meyeri* exposure was associated with alterations in microglial markers in the brain. An increased IBA1 signal was observed in *A. meyeri*‐treated mice compared to controls (Figure S3), without regional specificity (Figure S4). Similar but less pronounced changes were observed in the *A. odontolyticus* group in both RNA and protein levels (Figure S3). Given that microglial populations were not reliably captured in the scRNA‐seq dataset in the study, these observations are presented as supportive findings only.

Anxiety has been associated with increased neuroinflammation, including alterations in interleukin‐1β (IL‐1β), Tumor necrosis factor (TNF‐α), and IL‐6, as an immune response [13]. Given the reported association between anxiety and neuroinflammation, we next examined whether chronic oral *A. meyeri* inoculation was associated with changes in selected systemic or CNS inflammatory markers. To do so, we collected serum, saliva, gut, and brain from mice at the end of the study. No significant changes were observed in the three inflammatory cytokines examined in the brain, saliva, serum, or gut compared with vehicle‐treated controls (Figure 2). Unexpectedly, brain IL‐1β levels following *A. meyeri* exposure showed a decrease compared to the *A. odontolyticus* group and a trending decrease versus *N. elongata* and vehicle controls (Figure 2).

**FIGURE 2:**
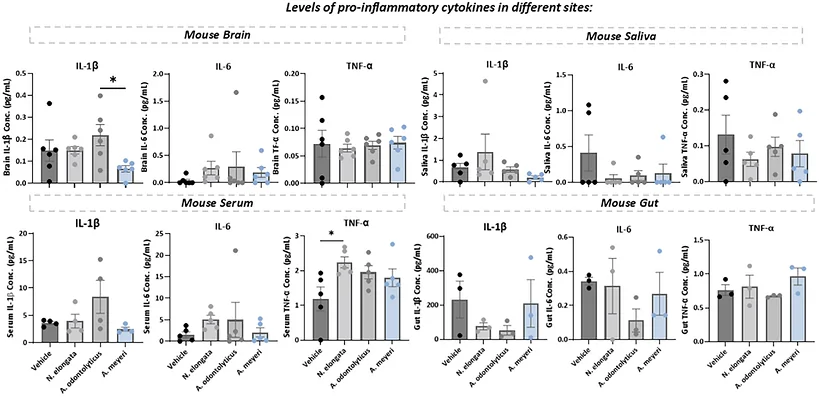
Chronic oral inoculation of *A. meyeri* is not associated with changes in selected pro‐inflammatory cytokine levels across brain and peripheral tissues in mice. Protein levels of the selected pro‐inflammatory cytokines IL‐1β, IL‐6, and TNF‐α were measured in the mouse brain, saliva, serum, and gut. No significant differences were observed between the *A. meyeri*‐treated group and vehicle controls across these compartments, although brain IL‐1β levels were reduced in the *A. meyeri*‐treated group compared to the *A. odontolyticus*‐treated group. Data are presented as mean ± SEM. Brain samples: *n* = 6 per group; serum and saliva samples: *n* = 4–6 per group, with samples below the assay detection limit excluded from quantitative analysis; gut samples: *n* = 3 randomly selected biological samples per group. ^*^ *p* < 0.05; one‐way ANOVA, followed by Tukey's multiple comparison test.

### 2.3 Chronic Oral *A. meyeri* Exposure Alters Mitochondrial‐ and GABA‐Related Gene Expression in the Brain

To study the exploratory transcriptomic landscape of various cell types in the brains of mice treated with *A. meyeri* (target oral bacterium), *A. odontolyticus*, *N. elongata* (bacterial controls), and vehicle control, the scRNA‐seq (10X Genomics) technology was used (Figure 3A). Nuclei were isolated from the intact pooled whole half‐brains of three mice from each experimental group, generating one integrated sample for each treatment condition. The estimated number of single cells obtained from each suspension was 14 000 to 20 000, whereas the target to capture was 3000–4000 nuclei in each integrated sample (Figure S5A). We identified 22 major cell types shown in different colors in *A. meyeri* vs. vehicle UMAP dimensional plots (Figure 3B), while the other two UMAPs (*A. meyeri* vs. *A. odontolyticus* and *A. meyeri* vs. *N. elongata*) and their subsequent downstream analysis are provided in Figures S5B–E. These included, Somatostatin+ Interneurons (*Sst*), Layer 6 Intratelencephalic (IT) Neurons (*L6 IT*), Layer 6 Intratelencephalic Neurons (Car3) (*L6 IT Car3*), Layer 5 Extratelencephalic (ET) Neurons (*L5 ET*) LAMP5+ GABAergic Interneurons (*Lamp5*), Parvalbumin+ Interneurons (*Pvalb*), Layer 2/3 Intratelencephalic (IT) Neurons (*L2/3 IT*), Layer 6 Corticothalamic (CT) Neurons (*L6 CT*), Layer 5 Intratelencephalic (IT) Neurons (*L5 IT*), Astrocytes (*Astro*), Oligodendrocytes (*Oligo*), Meis2+ GABAergic Interneurons (*Meis2*), Oligodendrocyte Precursor Cells (*OPC*), Pericytes (*Peri*), Vascular‐Associated Leptomeningeal Cells (*VLMC*), Endothelial Cells (*Endo*), Layer 5/6 Neocortical Projection Neurons (NP) (*L5/6 NP*), Layer 6b Neurons (*L6b*), Microglia and Paravascular Macrophages (PVM) (*Micro‐PVM*), Synuclein Gamma (SNCG)+ Neurons (*Sncg*), Sst+ Chodl+ Interneurons (*Sst Chodl*), VIP+ Interneurons (*Vip*). Our exploratory transcriptomic analysis showed that chronic oral *A. meyeri* exposure was associated with a greater number of genes showing increased or decreased relative expression in *L6IT, L6CT, L5ET, L5IT, L2/3IT* (glutamatergic excitatory pyramidal neurons), *Oligo* (glial cell; oligodendrocytes), and *Lamp5* (one of the several inhibitory GABAergic interneuron classes) compared to the other three study groups (Figure 3B,C, Figure S5). Although we included microglial activation markers in Figure S3, Micro‐PVM were not properly detected in the scRNA‐sSeq dataset due to technical limitations and thus were not specifically targeted here.

**FIGURE 3:**
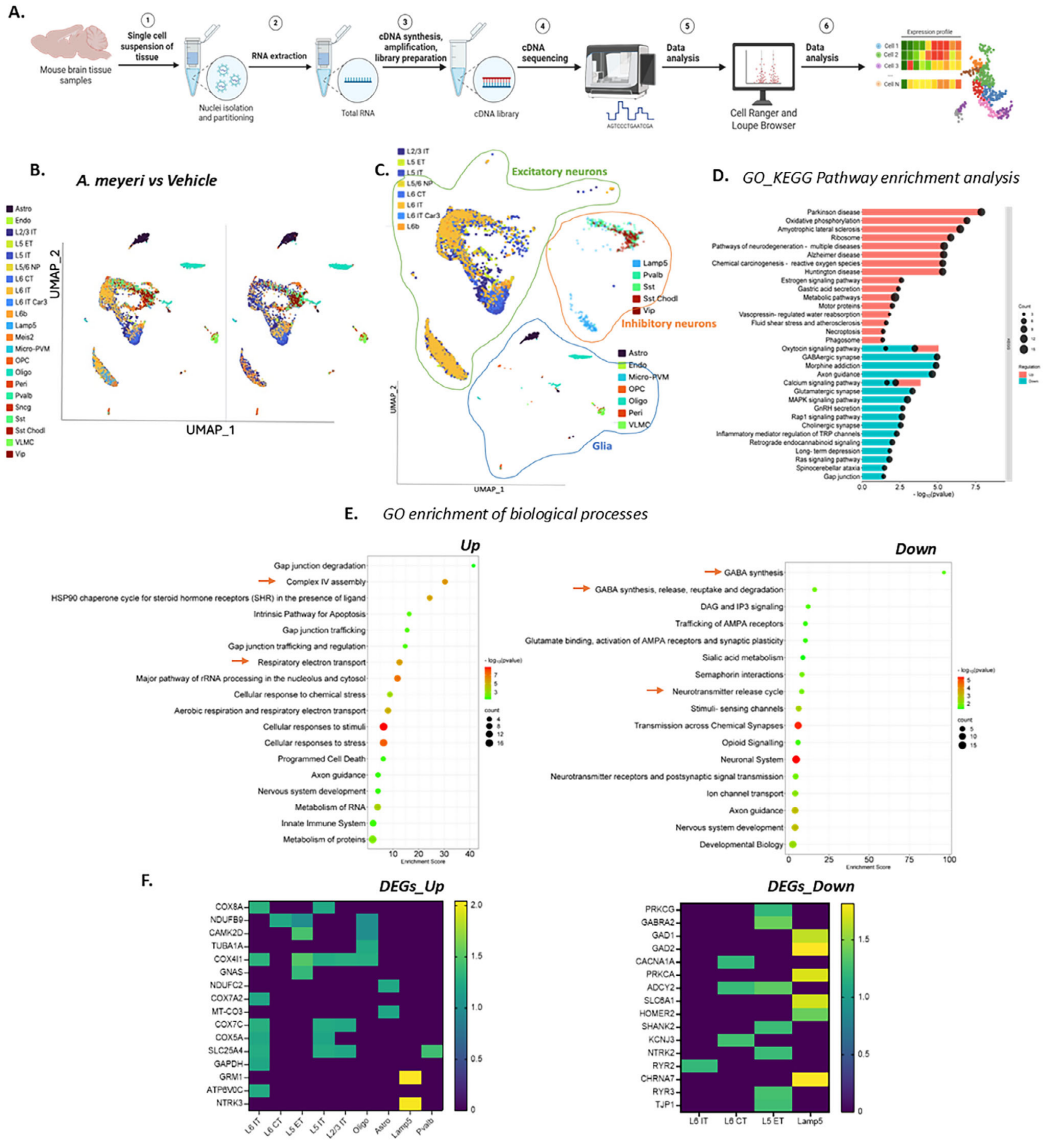
Chronic oral inoculation with *A. meyeri* is associated with broad transcriptional changes in neuronal cell types. (A) Schematic diagram illustrating the workflow for single‐cell RNA sequencing (scRNA‐seq) analysis of mouse brains. Whole half‐brains from three mice per experimental group were pooled to generate one integrated sample for each condition. (B) Uniform Manifold Approximation and Projection (UMAP) dimensional plots showing the integrated dataset split by treatment with *A. meyeri* versus vehicle control. Twenty‐two distinct cell types were identified by scRNA‐seq and are shown in different colors. (C) UMAP plot showing the classification of cell types into excitatory neurons, inhibitory neurons, and glial cells, along with their relative abundances and interactions. (D) KEGG pathway analysis of prioritized genes showing increased (pink) or decreased (blue) relative expression difference, with circle size indicating gene counts and color scale representing –log10 p‐values, which were used for exploratory pathway prioritization. (E) Gene ontology analysis of Reactome/biological processes using the DAVID annotation tool, displaying pathways prioritized from genes showing increased or decreased relative expression, enrichment scores [–log10(geometric mean of p‐values)], gene counts (circle size), and –log10 p‐values (color‐coded). These pathway‐level statistics were used as exploratory annotation and prioritization metrics. Pathways related to neurodegenerative disorders and mitochondrial processes, including oxidative phosphorylation/respiratory electron transport, were prioritized from genes showing increased relative expression. In contrast, pathways associated with GABAergic neurotransmission (synthesis, release, and reuptake) were prioritized from genes showing decreased relative expression in *A. meyeri*–treated mice compared with controls. (F) Heatmaps show prioritized genes with increased or decreased relative expression, based on average log_2_ fold‐change, across different cell types in each integrated dataset. These exploratory transcriptomic findings were subsequently evaluated by independent biochemical and functional validation experiments.

The horizontal stack bar plot was generated from KEGG pathway analysis and illustrates pathway‐level patterns derived from prioritized genes showing increased or decreased relative expression (Figure 3D). Neurodegeneration‐related and mitochondrial pathways were among those prioritized from genes showing increased relative expression, whereas G‐protein‐coupled receptor and GABAergic synapse pathways were prioritized from genes showing decreased relative expression in the *A. meyeri* group compared with the control groups (Figure 3D, Figure S5C). Next, we examined gene ontology enrichment of the biological processes in the same integrated dataset (Enrichment bubbles in Figure 3E). Enrichment score (ES) in the DAVID bioinformatics tool was used as an exploratory metric to prioritize relevant pathway annotations. The ES is derived from the geometric mean (on a log scale) of the p‐values associated with annotations within a given cluster. In the present analysis, these pathway‐level statistics were used for exploratory annotation and prioritization and were not interpreted as animal‐level statistical significance. Many biological processes were identified in the transcriptomic landscape following chronic oral treatment with *A. meyeri*. Mitochondrial‐related pathways and GABAergic neurotransmission were consistently prioritized from genes showing increased and decreased relative expression, respectively, across comparisons of the *A. meyeri*‐treated group with the other study groups (Figure 3E, Figure S5C). For instance, complex IV assembly, oxidative phosphorylation, and the respiratory electron transport chain are mostly flagged under the aspect of mitochondrial involvement. Similarly, gene ontology analysis of biological processes (BP) prioritized multiple components of GABAergic neurotransmission among genes showing decreased relative expression following oral *A. meyeri* exposure. This included genes involved in GABA biosynthesis, synaptic release, reuptake via transporters, and enzymatic degradation, suggesting potential alterations in inhibitory signaling within the CNS. Overall, these analyses identified an exploratory transcriptional signature characterized by increased relative expression of mitochondrial‐related genes together with decreased relative expression of GABA‐related pathways following oral *A. meyeri* exposure.

To further examine the cell‐type‐specific transcriptional patterns associated with *A. meyeri* administration, we examined the distribution of prioritized genes across the 22 cell types. Heatmaps of genes showing increased or decreased relative expression revealed that the majority of these transcriptional changes were concentrated in excitatory neuronal subtypes (*L5 ET* and *IT, L6 CT* and *IT, L2/3 IT*) and Lamp5‐expressing GABAergic inhibitory interneurons, with additional but limited involvement of oligodendrocytes, astrocytes, and Meis2+ GABAergic interneurons (Figure 3F). Most of the mitochondrial markers or stress genes (e.g., COX genes, NDUF, SLC25 family) showed increased relative expression in the excitatory neuronal subtypes in the *A. meyeri*‐treated group, with similar patterns observed for some genes in oligodendrocytes and astrocytes.

GAD1 and GAD2 (glutamate decarboxylase 1 and 2) are rate‐limiting enzymes for neuronal GABA synthesis. GAD1 is primarily involved in cytoplasmic GABA synthesis for metabolic purposes and extrasynaptic release, while GAD2 is more involved in regulating the vesicular pool of GABA for synaptic release [14]. In GABAergic cells, SLC6A1 encodes the GABA transporter 1 (GAT‐1), which is crucial for clearing GABA from the extracellular space [15]. In the heatmap of prioritized genes showing decreased relative expression for *A. meyeri* compared to vehicle control (Figure 3F), GAD1, GAD2, and SLC6A1 (or GAT‐1) showed decreased relative expression in Lamp5 interneurons. GABRA2, which encodes the α2 subunit of the postsynaptic GABA‐A receptor (postsynaptic), also showed decreased relative expression in Layer 5 ET (excitatory glutamatergic cell type). Together, these patterns are consistent with reduced transcriptional capacity for GABA synthesis and reuptake, suggesting potential reductions in inhibitory signaling. Layer 5 ET neurons integrate inhibitory input from local interneurons (e.g., LAMP5, PV, SST cells), and reduced GABRA2 expression suggests a diminished transcriptional capacity to respond to GABAergic input. These exploratory transcriptomic findings are consistent with alterations in mitochondrial and GABA‐related pathways. Selected mitochondrial‐ and GABA‐associated targets identified from this transcriptomic screen were subsequently examined using independent protein and biochemical validation (Figures 5 and 6). However, additional functional studies in brain tissues will be required to establish the causal relationship between these molecular changes and the observed behavioral phenotype.

### 2.4 Candidate Metabolites Associated with Oral‐Brain Communication in the Absence of Detectable Bacterial DNA, RNA, or Peptidoglycan (PGN)

To further investigate the potential mechanism underlying the association between oral *A. meyeri* exposure and the observed neurobehavioral alterations, we assessed potential mediators of oral‐to‐brain communication, including bacterial DNA, RNA, cell wall/membrane products (e.g., PGN), or metabolites, which may translocate from the oral cavity to the brain. First, no detectable *A. meyeri‐*specific RNA signal was found in any region of the brain using an RNAScope‐based *A. meyeri*‐specific probe in the sagittal mouse brain FFPE sections (Figure 4A). We also tested specific probes for *A. odontolyticus* and *N. elongata*, and no signal was detected in the control microbiome‐treated mouse brains as well (Figure S6A). Second, we conducted qPCR using specific *A. meyeri* primers, but no bacterial DNA was detected; purified bacterial culture DNA was used as a positive control (Figure 4B). Bacterial‐specific transgenes were included in the scRNA‐seq study; consistently, no transgenes of *A. meyeri* (or two other microbiomes) were found in the mice brains (Figure 4C). After confirming the absence of bacterial DNA and RNA in the brain, we evaluated bacterial PGN in the brain using the Fujifilm Wako chemicals kit. Bacterial PGN was undetected in the brain lysates but was detected in the positive control using the lysed bacteria (Figure 4D).

**FIGURE 4:**
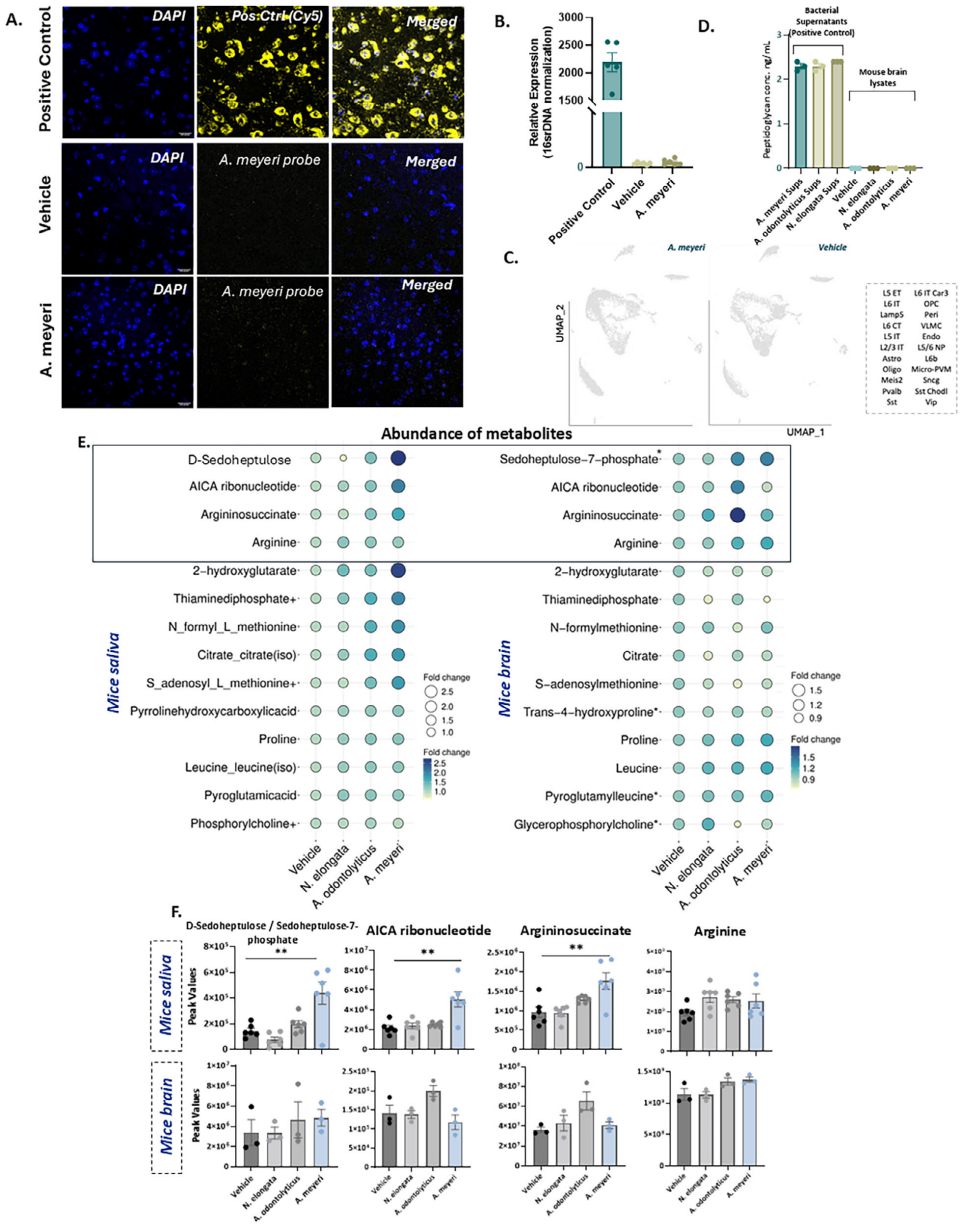
No evidence of oral bacterial translocation and abundance patterns of candidate metabolites. (A) RNAScope in situ hybridization of sagittal mouse brain sections (5 µm FFPE). Fluorescent imaging showed no detectable *A. meyeri* RNA signal (yellow, Cy5 channel). Manufacturer‐provided positive probe (UBC, a housekeeping gene) confirmed assay performance and specificity. Images were acquired at 40× magnification on a Zeiss LSM 510 Meta confocal microscope (1024 × 1024; *n* = 4). (B) Quantitative PCR analysis of brain lysates using *A. meyeri*–specific primers revealed no detectable bacterial DNA. Standard curves generated from serial dilutions of purified bacterial DNA served as positive controls for assay validation. (C) Targeted analysis using organism‐specific transgene reference sequences in the scRNA‐seq dataset (NCBI RefSeq: NR_029286.1) detected no *A. meyeri*‐specific RNA reads, providing no evidence of bacterial translocation to the brain. (D) Peptidoglycan levels were determined in the brain lysates using the Silkworm larvae assay kit. Pure bacterial lysates were included as positive controls to verify assay performance. (E) Metabolomic profiling is illustrated by balloon plots showing a biologically prioritized cross‐compartment subset based on abundance patterns and potential biological relevance. The boxed region highlights the four candidate metabolites selected for further evaluation and shown individually in panel F. Asterisks indicate brain metabolites representing related but non‐identical chemical features to the corresponding metabolites detected in saliva. (F) Normalized relative peak intensities of the four candidate metabolites are shown across saliva and brain in all three bacterial treatment groups. For metabolomic analyses, saliva samples were n = 6 per group and whole‐brain samples were *n* = 3 per group. Metabolites were analyzed using ordinary one‐way ANOVA followed by planned treatment‐versus‐vehicle contrasts using the pooled ANOVA residual variance, with Benjamini‐Hochberg FDR correction applied across the full metabolite comparison family within each contrast. Data are presented as mean ± SEM. ^**^ indicates FDR‐adjusted significance (*q* < 0.01); none of the four candidate brain metabolites remained significant after FDR correction.

Microbiome dysbiosis occurs in many physiological conditions, such as gut‐related health issues, and has previously been associated with altered metabolite levels, for instance, short‐chain fatty acids or indoles [16]. Microbial metabolites have been proposed as one potential means of host‐microbe communication. To investigate this potential oral‐to‐brain mechanism, we conducted a metabolomics study to examine metabolic changes in both the oral cavity and the brain. Untargeted metabolomic profiling was performed on oral swabs and whole‐brain samples (brain: *n* = 3 per group), and metabolite abundance is reported as normalized relative peak intensities. A total of 211 metabolites were detected in the oral swabs, while around 919 metabolites (860 with known chemical identity and 59 unknown) were detected in the mouse brains. Following Benjamini‐Hochberg false discovery rate correction across all 211 detected oral metabolites, 67 metabolites differed between the A. meyeri and vehicle groups (q < 0.05), of which 64 showed increased abundance and three showed decreased abundance. We subsequently examined metabolites detected across oral and brain datasets and prioritized a subset based on their cross‐compartment abundance patterns and potential biological relevance (Figure 4E). From this integrated comparison, we prioritized four candidate metabolites for further evaluation, namely sedoheptulose‐7‐phosphate, 5‐aminoimidazole‐4‐carboxamide (AICA) ribonucleotide, arginine, and argininosuccinate (Figure 4F). In the oral cavity, D‐sedoheptulose (2.47‐fold, p = 0.00015, q = 0.00242), AICA ribonucleotide (2.35‐fold, p = 0.00012, q = 0.00239), and argininosuccinate (1.83‐fold, p = 0.00040, q = 0.00471) remained significantly increased following *A. meyeri* exposure after FDR correction, whereas oral arginine did not differ significantly from vehicle (1.30‐fold, p = 0.1078, q = 0.1835). In the brain metabolomic dataset, none of the four candidate metabolites remained significant after Benjamini‐Hochberg correction across the full metabolite comparison family. Brain arginine showed a modest increase in the *A. meyeri* group (fold change = 1.21, p = 0.0316, q = 0.2905), whereas argininosuccinate showed its largest abundance difference in the *A. odontolyticus* group (fold change = 1.80, p = 0.0123, q = 0.2435). AICA ribonucleotide likewise showed its largest increase in the *A. odontolyticus* group (fold change = 1.41, p = 0.0409, q = 0.3242), whereas sedoheptulose‐7‐phosphate showed modest abundance differences in the brain (AM: fold change = 1.43, p = 0.4184, q = 0.7072; AO: fold change = 1.37, p = 0.4844, q = 0.8045). These brain metabolite patterns were therefore considered exploratory and were used for candidate prioritization rather than interpreted as statistically confirmed metabolite alterations.

Together, these findings indicate a tissue‐specific pattern of arginine‐related metabolites rather than a uniform increase across the oral cavity and brain. Oral argininosuccinate was significantly increased following *A. meyeri* exposure after FDR correction, whereas oral arginine remained unchanged. In the brain, arginine showed modest abundance differences in the *Actinomyces*‐treated groups, while argininosuccinate showed its largest increase following *A. odontolyticus* exposure; however, neither metabolite remained significant after multiple‐testing correction. These patterns suggest compartment‐specific regulation of arginine‐related metabolism. Furthermore, bacterial supernatants were screened to determine whether these metabolites could be produced by the Actinomyces species under the culture conditions examined. Arginine and argininosuccinate were detected in the culture supernatants of both *A. meyeri* and *A. odontolyticus*, indicating that both species can produce these metabolites in vitro (Figure S6B). AICA ribonucleotide showed its largest brain abundance difference in the *A. odontolyticus* group but was not detected as a bacterial product under the culture conditions examined. D‐sedoheptulose was significantly increased in the oral cavity following *A. meyeri* exposure, whereas the related metabolite sedoheptulose‐7‐phosphate was detected in the brain and in the culture supernatants of *A. odontolyticus*, but not *A. meyeri*. Based on the integrated metabolomic and bacterial‐supernatant observations, we prioritized arginine and argininosuccinate as candidate metabolites for subsequent functional studies because of their differential abundance and established links to mitochondrial metabolism and neurotransmitter biology. Although the metabolite profiles differed between oral and brain compartments, these two metabolites were selected as candidates for functional evaluation rather than as evidence of a direct oral‐to‐brain transfer pathway.

### 2.5 Chronic Oral *A. eyeri*‐Associated Metabolites Alter Mitochondrial Function and ROS Production in N2a Cells

As shown in Figure 3, oxidative phosphorylation and the respiratory electron transport were among the pathways prioritized from genes showing increased relative expression. To visualize these mitochondrial transcriptional alterations associated with *A. meyeri* treatment, we mapped prioritized genes showing increased relative expression onto a schematic of the electron transport chain (ETC) (Figure 5A). Genes encoding subunits of complex I (NDUFB9, NDUFC2) and complex IV/cytochrome c oxidase (COX4I1, COX5A, COX7A2, COX7C, COX8A), together with mitochondrially encoded MT‐CO3, showed increased relative expression. In addition, SLC25A4, an inner mitochondrial membrane carrier that mediates ADP/ATP exchange but does not directly belong to any respiratory complex, showed increased relative expression [17]. Although ATPV06, a subunit of the vacuolar ATPase, also showed increased relative expression, it was not included in the schematic because it localizes primarily to lysosomal membranes rather than mitochondria [18]. Together, these transcriptomic changes suggest coordinated remodeling of mitochondrial respiratory chain and nucleotide transport components. From the transcriptomic screen, we selected COX4I1 and SLC25A4 for validation based on their functional relevance to mitochondrial activity. COX4I1 encodes a nuclear subunit of cytochrome c oxidase (Complex IV), the terminal electron acceptor in the respiratory chain, whereas SLC25A4 encodes the adenine nucleotide translocase (ANT1), which exchanges ADP and ATP across the inner mitochondrial membrane and is critical for bioenergetic homeostasis [17]. The UMAP plots illustrate the increased relative expression of these genes mostly in the excitatory neurons (L2/3IT, L5, L6 subtypes) and oligodendrocytes (Figure 5B). Western blotting confirmed a significant increase in SLC25A4 protein expression in the *A. meyeri*‐treated group compared to vehicle controls, consistent with the transcriptomic findings; COX4I1 protein levels showed only a trend toward increased expression (Figure 5C,D). These results support SLC25A4 as a robust marker of mitochondrial remodeling in response to *A. meyeri*.

**FIGURE 5:**
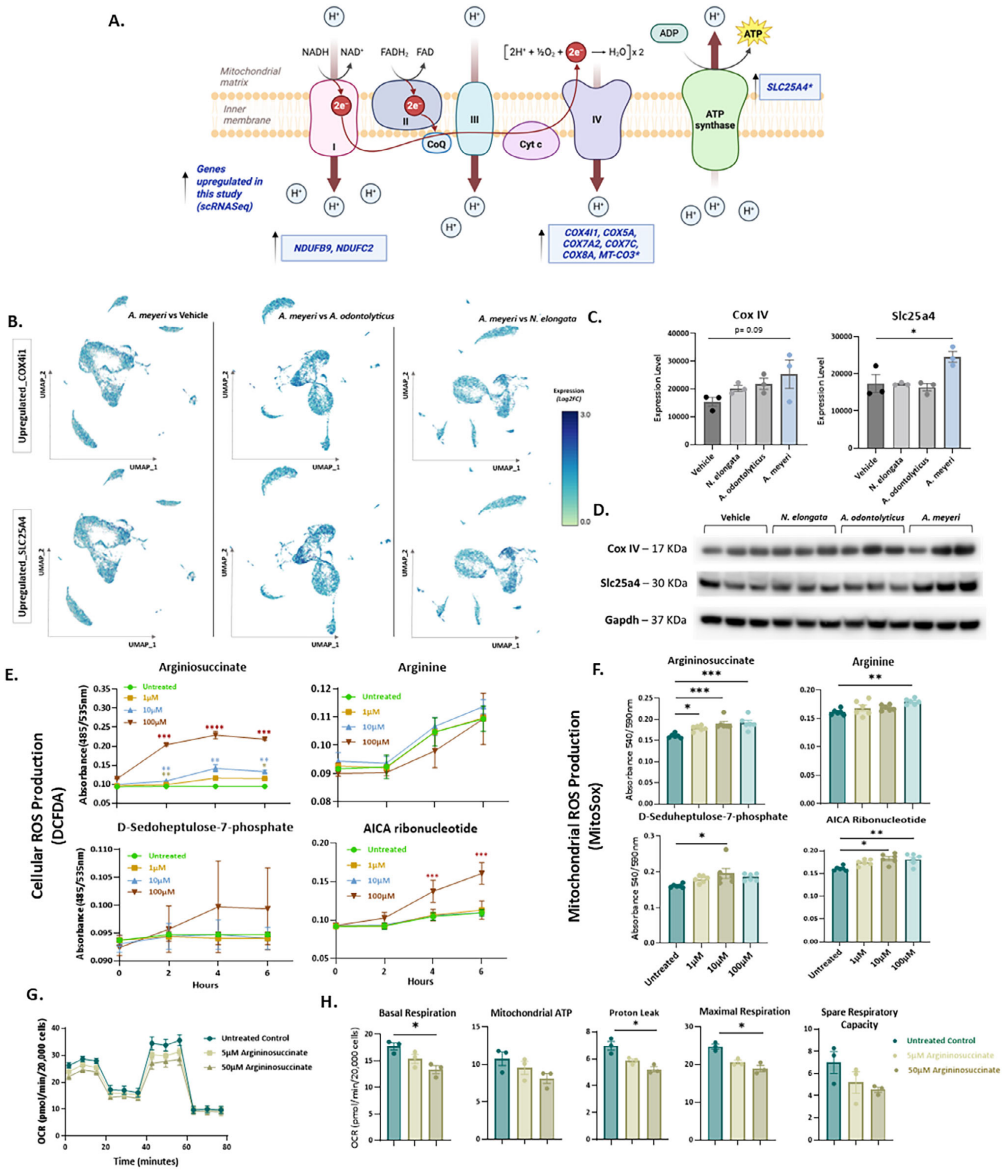
Chronic oral inoculation with ***A. meyeri*** is associated with mitochondrial remodeling supported by transcriptomic, proteomic, and metabolite‐based functional assays. (A) Schematic highlighting prioritized genes showing increased relative expression in mitochondrial respiratory complexes I and IV (with MT‐CO3 encoded by mitochondrial DNA, others by nuclear DNA) and SLC25A4 (inner membrane ADP/ATP carrier). These transcriptomic changes are consistent with mitochondrial remodeling and may reflect a compensatory response to altered cellular energy demands or stress. (B) UMAP plots from the exploratory scRNA‐seq analysis showing two shortlisted mitochondrial‐related genes, COX4I1, a nuclear‐encoded subunit of complex IV (cytochrome c oxidase) in the electron transport chain (ETC), and SLC25A4 (or ANT1), an ADP/ATP translocator in the inner mitochondrial membrane (not part of the ETC). These exploratory transcriptomic analyses showed increased relative expression of COX4I1 and SLC25A4 in excitatory neurons (L2/3 IT, L5, L6 subtypes) and oligodendrocytes in the *A. meyeri* condition. (C,D) Western blot analysis revealed a trend toward increased COX4I1 expression and a significant increase in SLC25A4 protein, providing independent protein‐level support for the exploratory transcriptomic observations. (E,F) Cellular ROS (DCFDA/H2DCFDA) and mitochondrial ROS (MitoSOX) assays in N2a cells treated with candidate metabolites (arginine, argininosuccinate, sedoheptulose‐7‐phosphate, AICA ribonucleotide; 1, 10, and 100 µm). Argininosuccinate treatment was associated with a dose‐dependent increase in cellular and mitochondrial ROS. (G) Oxygen consumption rate (OCR) was measured in N2a cells treated with two concentrations of argininosuccinate under mitochondrial stress conditions (oligomycin, FCCP, and rotenone/antimycin A). (H) OCR analyses demonstrated altered basal and maximal respiration, mitochondrial ATP production, proton leak, and spare respiratory capacity following argininosuccinate treatment. DCFDA, MitoSOX, and Seahorse assays were performed in three independent experiments (n = 3), with three, six, and ten technical replicate wells per treatment condition, respectively. Data are presented as mean ± SEM. ^*^ *p* < 0.05, ^**^ *p* < 0.01, ^***^ *p* < 0.001; one‐way ANOVA or mixed‐effects two‐way ANOVA with Tukey's post hoc test.

Because our transcriptomic and protein analyses identified mitochondrial remodeling signatures (Figure 5A‐‐D), we next examined whether the shortlisted metabolites altered by *Actinomyces species* exposure (Figure 4E,F) could themselves perturb mitochondrial homeostasis. We treated Neuro2a cells with various concentrations (1, 10, and 100 µm) of the metabolite and assessed their effects on cellular and mitochondrial reactive oxygen species (ROS) production, as well as mitochondrial respiration. Using the DCFDA assay (Figure 5E), argininosuccinate elicited a dose‐dependent increase in total cellular ROS, whereas AICA ribonucleotide induced an increase only at the highest concentration (100 µm) in a time‐dependent manner. MitoSOX analysis (Figure 5F) further confirmed that argininosuccinate produced a robust, dose‐dependent elevation in mitochondrial ROS, with additional effects observed for arginine (100 µm) and AICA ribonucleotide (10 and 100 µm). In contrast, sedoheptulose‐7‐phosphate did not affect total ROS but caused a modest increase in mitochondrial ROS at 10 µm. Given its consistent effects across both ROS assays, we next examined argininosuccinate in a mitochondrial stress test using the Seahorse XF analyzer (Figure 5G,H). Treatment with argininosuccinate (5 and 50 µm) produced dose‐dependent changes in mitochondrial respiration, with significant reductions in basal respiration, proton leak, and maximal respiration at 50 µm compared with untreated controls. Together, these findings demonstrate that argininosuccinate is capable of altering mitochondrial respiration and ROS production in neuronal‐like cells in vitro, supporting its prioritization as a candidate metabolite for further investigation in the context of oral Actinomyces‐associated neurobiological alteration.

### 2.6 Chronic Oral *A. meyeri* Exposure is Associated with Reduced GABA‐Related Markers and GABA Levels in the Brain

Chronic oral inoculation with *A. meyeri* was associated with behavioral alterations (Figure 1) and transcriptional changes in mitochondrial‐related pathways (Figures 3, 4, 5). Given that mitochondria provide intermediates and essential metabolic precursors for neurotransmitter synthesis, including glutamate and GABA [19, 20], we next examined whether markers of GABAergic pathways were altered. As shown in Figure 3, scRNA‐seq analysis identified decreased relative expression of genes associated with GABAergic pathways in the *A. meyeri* group. Moreover, transcriptomic analysis (Figure 6A) revealed decreased relative expression of genes involved in multiple components of GABA signaling, including GAD1 and GAD2 (GABA synthesis), SLC6A1 (GAT1; reuptake), CACNA1A (presynaptic release), and postsynaptic components such as GABRA2 and PRKCG/PRKCA [21]. The UMAP plots demonstrated decreased relative expression of GAD1, GAD2, and SLC6A1 in GABAergic neurons (Lamp5‐positive) and astrocytes, with the *A. meyeri*‐treated mice showing the most pronounced relative expression differences (Figure 6C). Consistent with these transcriptomic observations, whole‐brain GABA levels (Figure 6B) were significantly reduced in the *A. meyeri* group relative to other microbiome groups. Western blot analysis demonstrated decreased protein expression of GAD1 and GAD2 in both *Actinomyces*‐treated groups, whereas GAT1 showed a downward trend (Figure 6D,E). Together, these findings indicate coordinated changes in GABA‐related molecular markers and neurotransmitter levels following *A. meyeri* exposure.

**FIGURE 6:**
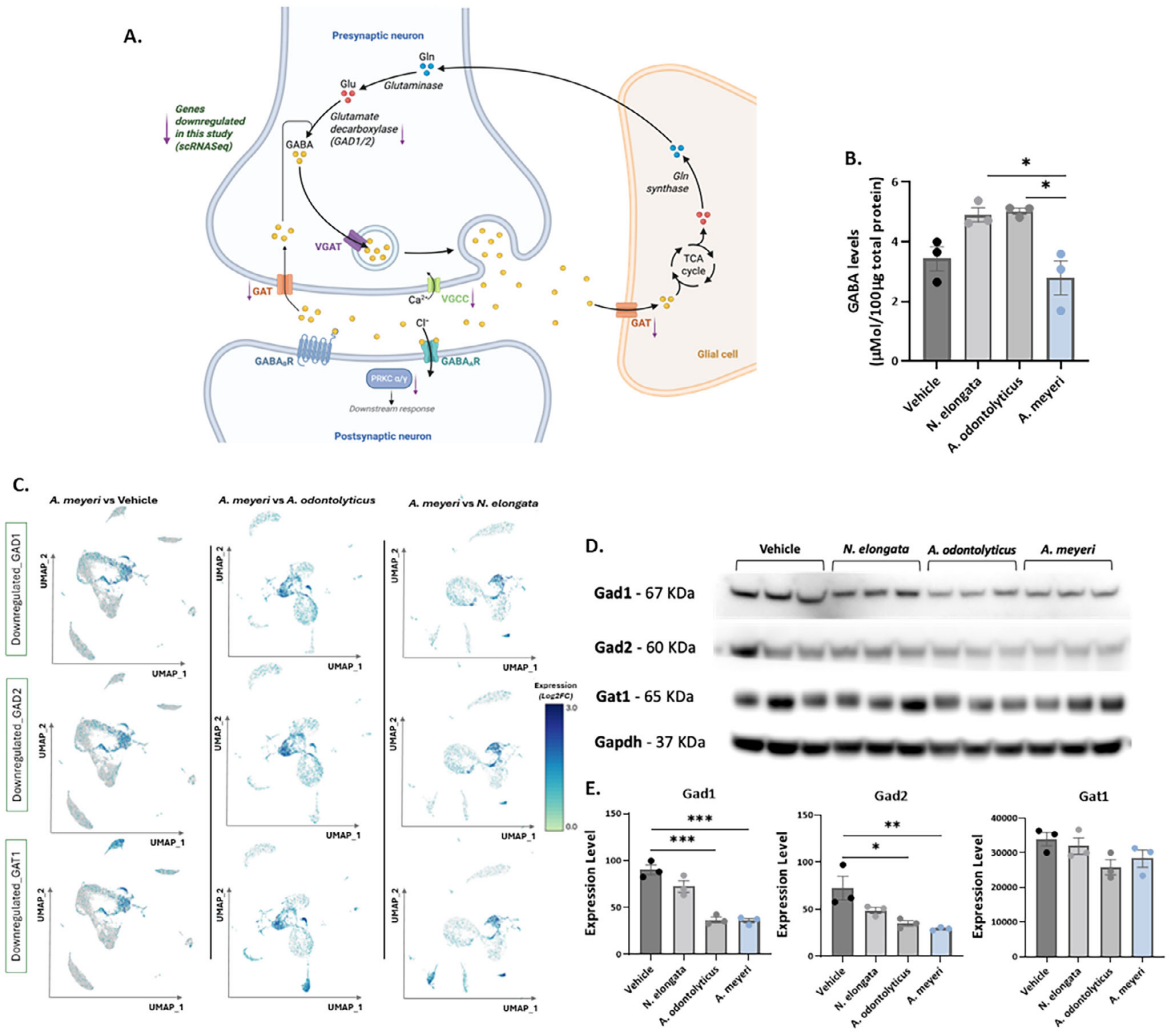
Chronic oral inoculation with *A. meyeri* is associated with altered GABAergic pathways. (A) Schematic illustrating the exploratory transcriptomic changes associated with GABAergic pathways. Reduced transcription of genes involved in GABA synthesis (GAD1/GAD2), reuptake (SLC6A1/GAT1), presynaptic release (CACNA1A), and postsynaptic signaling (GABRA2, PRKCG, and PRKCA) was observed following oral *A. meyeri* exposure. (B) Quantification of whole‐brain GABA levels in mouse brain homogenates, after normalizing to the total protein content in each sample, revealed significant reductions in *A. meyeri*–treated mice compared with other microbiome groups. (C) UMAP plots from exploratory scRNA‐seq analysis (between‐group comparison with *A. meyeri* group as reference) identified reduced relative expression of three genes in GABAergic neurons (Lamp5) and astrocytes: GAD1 (glutamate decarboxylase 67), GAD2 (glutamate decarboxylase 65), and SLC6A1 (GAT1). (D,E) Western blot analysis supported the transcriptomic observations by demonstrating decreased GAD1 and GAD2 protein levels, with a trend toward reduced GAT1 expression in both Actinomyces groups relative to vehicle controls. Data are presented as mean ± SEM. For GABA quantification and Western blot analyses, *n* = 3 biological samples per group. ^*^ *p* < 0.05, ^**^ *p* < 0.01, ^***^ *p* < 0.001; one‐way ANOVA with Tukey's post hoc test.

Although *A. odontolyticus* also showed modest reductions in GAD1 and GAD2 protein levels and a trend toward reduced GAT1, the exploratory scRNA‐seq analysis identified more pronounced and consistent transcriptional changes in the *A. meyeri* group (Figure 6A,C). Behavioral assays similarly showed anxiety‐like phenotypes most prominently in *A. meyeri*‐treated mice, with only a non‐significant trend observed in *A. odontolyticus* (Figure 1). While mitochondrial‐related gene expression changes were enriched in excitatory neuronal populations (Figure 5), the observed alterations in GABAergic markers may reflect network‐level interactions between excitatory and inhibitory circuits, where changes in excitatory neuronal states may influence inhibitory signaling dynamics. However, additional region‐ and cell‐type‐specific functional studies will be required to define the mechanistic relationship between these transcriptional and biochemical alterations and their contribution to neuronal circuit function.

## 3 Discussion

Cannabis and other substances of abuse are widely used and have been associated with an increased risk of neuropsychiatric conditions [22, 23], although the underlying biological mechanisms remain incompletely understood. In our previous work, we reported enrichment of Actinomyces species in the oral microbiome of chronic cannabis users [4]. Building on these findings, we investigated whether oral exposure to *Actinomyces*, in the absence of cannabis or its psychoactive components, could influence brain function and behavior. In the present study, chronic oral inoculation of *A. meyeri* in wild‐type C57BL/6 mice was associated with anxiety‐like behavior without significant alterations in canonical proinflammatory cytokines (IL‐1β, IL‐6, TNF‐α). Transcriptomic and biochemical analyses identified coordinated changes in mitochondrial‐related gene expression and GABA‐associated pathways. Notably, no evidence of bacterial or peptidoglycan translocation to the brain was detected. Instead, alterations in metabolites related to the arginine–argininosuccinate axis were observed, and selected metabolites altered mitochondrial function and ROS production in neuronal‐like cells in vitro. These findings nominate arginine and argininosuccinate as candidates for further investigation as potential mediators of oral microbiome‐brain communication.

### 3.1 Oral *A. meyeri* Exposure is Associated with Anxiety‐Like Behavior

The oral microbiome not only plays a role in oral health but also can affect brain functions through the trigeminal nerve or olfactory system connections, as well as indirectly through the oral‐gut‐brain axis [24]. The gastrointestinal tract contains a variety of bacteria whose metabolic products impact several physiological processes, such as stress, anxiety, depression, bowel diseases, and others [25]. In contrast, the role of the oral microbiome and its impact on neurophysiological functions has only recently begun to emerge [26]. The genus Actinomyces, predominantly anaerobic or facultative‐anaerobic, is not abundant in the gastrointestinal tract as compared with the oral cavity [5]. In our previous investigations, we consistently failed to detect *A. meyeri* in the gut of mice orally inoculated with this bacterium for six months [4]. This may be attributed to unfavorable gut conditions, including hypoxic gradients, limited nutrient availability, and competition with established microbial communities [27]. Collectively, our findings support the possibility that oral‐brain communication involves candidate microbial metabolites rather than gut‐derived mechanisms, although direct evidence that these metabolites are transferred from the oral cavity to the brain remains to be established. Previous studies have shown that gut microbiota influence brain function through the production of neuroactive metabolites, including GABA and other small molecules capable of modulating neuronal signaling, behavior, and neurodevelopment [28, 29, 30]. These studies have largely focused on the gut microbiome, whereas comparatively little is known about whether the oral microbiome exerts similar effects independent of gut colonization. Our findings extend this emerging concept by identifying candidate metabolites associated with chronic oral *A. meyeri* exposure and molecular alterations consistent with oral‐brain communication in the absence of detectable bacterial translocation.

Behaviorally, the anxiety‐like phenotype observed in this study was most consistently reflected in the open‐field test, in which *A. meyeri*‐treated mice exhibited increased peripheral exploration and reduced center occupancy without significant differences in total distance traveled. Although spontaneous home‐cage activity, measured by infrared beam breaks, was increased following bacterial treatment, this assay evaluates ambulatory activity in a familiar environment rather than anxiety‐related behavior. In contrast, the OFT assesses exploratory behavior in a novel environment, where total distance traveled did not differ among groups. Moreover, analysis using total OFT distance as a covariate showed that the group effect on center time remained significant after accounting for locomotor activity, whereas the adjusted effect on periphery time was attenuated. Together, these findings suggest that locomotor activity may contribute to some aspects of the OFT phenotype but does not fully account for the reduced center occupancy. The increased home‐cage activity across bacterial treatment groups, including *N. elongata*, nevertheless indicates that bacterial exposure also influenced spontaneous ambulatory activity. The elevated plus‐maze showed a similar directional pattern, although the effect was less pronounced than that observed in the open‐field test. Overall, these behavioral findings are consistent with altered anxiety‐related exploratory behavior, although replication in an independent cohort will be important to further strengthen confidence in these findings.

Consistent with the cytokine profile observed in this study, levels of proinflammatory cytokines (IL‐1β, IL‐6, and TNF‐α) remained unchanged across brain and peripheral tissues from their respective vehicle controls (Figure 2), indicating that the observed behavioral alterations were not accompanied by changes in the proinflammatory cytokines examined. Microglial markers were also evaluated (Figure S3); however, these findings were not further explored because Micro‐PVM populations were insufficiently represented in the scRNA‐seq dataset for meaningful transcriptomic analysis. Together, these results support the possibility that oral enrichment of *A. meyeri* is associated with behavioral alterations through oral‐brain communication pathways, potentially involving soluble microbial products, including candidate metabolites, rather than gut‐derived mechanisms.

### 3.2 Oral *A*.m*eyeri* Exposure is Associated with Coordinated Changes in Mitochondrial‐ and GABA‐Related Pathways

Our single‐cell transcriptomic analysis identified an exploratory transcriptional signature characterized by increased relative expression of mitochondrial‐related genes and decreased relative expression of GABA‐related genes associated with GABA‐related pathways following chronic oral *A. meyeri* exposure. Mitochondrial hyperactivity is a newly recognized factor in neurodegenerative diseases like Parkinson's [31]. Rather than a late‐stage decline, overactive mitochondria may trigger early neuronal damage by increasing oxidative stress and disrupting energy balance, fueling a cycle of degeneration [31]. The increased relative expression of mitochondrial genes, particularly within excitatory pyramidal neurons in our study, is consistent with increased mitochondrial activity associated with oxidative stress and neuronal vulnerability in CNS disorders [32, 33]. Importantly, selected mitochondrial‐associated targets identified in the transcriptomic screen were subsequently supported by protein‐level validation, strengthening the association between the observed transcriptional changes and mitochondrial remodeling. Interestingly, although the exploratory transcriptomic analyses identified increased relative expression of mitochondrial‐related genes, exposure of Neuro2a cells to the candidate metabolite argininosuccinate reduced mitochondrial respiration in the Seahorse assay. These findings are not necessarily contradictory. Increased expression of mitochondrial‐associated genes may represent an adaptive or compensatory response to altered cellular energy demands or oxidative stress, whereas the Seahorse assay reflects functional mitochondrial respiration following acute metabolite exposure in vitro. Together, these complementary observations support mitochondrial remodeling rather than uniformly enhanced or impaired mitochondrial functions. Consistent with this concept, increased expression of SLC25A18 (a mitochondrial glutamate carrier) has recently been associated with Alzheimer's disease (AD) through transcriptomic analyses of human brain tissue and subsequent validation in animal models of AD and Neuro2A cells [34].

In parallel, decreased relative expression of GAD1, GAD2, and SLC6A1 in Lamp5 interneurons, along with reduced GABRA2 expression in Layer 5 excitatory neurons, is consistent with reduced transcriptional capacity for GABA synthesis, reuptake, and postsynaptic responsiveness. These coordinated alterations may contribute to reduced inhibitory tone and a shift in excitatory‐inhibitory balance. Such an imbalance is a well‐recognized mechanism underlying anxiety, epilepsy, and autism spectrum disorders [35, 36, 37, 38]. These transcriptomic observations were further supported by reduced brain GABA levels and decreased expression of selected GABA‐related proteins, strengthening the association between the transcriptomic findings and altered inhibitory signaling. Importantly, these exploratory transcriptomic patterns are consistent with the anxiety‐like phenotype observed in our behavioral assays and support a potential association between oral dysbiosis, mitochondrial remodeling, and altered GABAergic pathways. Although these complementary transcriptomic, protein, biochemical, and in vitro functional data support a coherent biological association, they do not directly demonstrate mitochondrial dysfunction or impaired inhibitory neurotransmission within specific brain regions or neuronal circuits. A limitation of the scRNA‐seq analysis is that brains from three mice per group were pooled into a single sample per condition, precluding animal‐level statistical inference; therefore, these transcriptomic findings should be interpreted as exploratory and hypothesis‐generating. Future studies incorporating region‐ and cell‐type‐specific mitochondrial functional assays, quantitative glutamate and GABA measurements, electrophysiological recordings, and functional rescue approaches will be required to establish the causal relationship between these molecular alterations and anxiety‐like behavior. Interestingly, comparison with bacterial controls revealed a genus‐specific pattern. *A. meyeri* and *A. odontolyticus* shared several candidate metabolites associated with mitochondrial metabolism, although the exploratory scRNA‐seq analysis showed more pronounced transcriptional patterns in *A. meyeri*, whereas *A. odontolyticus* frequently showed changes in the same direction with generally weaker effects. This was particularly evident when integrating the exploratory scRNA‐seq findings with subsequent protein‐level validation, which demonstrated reductions in GAD1, GAD2, and SLC6A1, as well as increases in COX‐IV (Cox4i1) and SLC25A4 expression, that were most pronounced in the *A. meyeri* group. These findings suggest that the members of the Actinomyces genus may differently influence mitochondrial‐ and GABA‐related pathways, potentially through candidate microbial metabolites, with *A. meyeri* eliciting a more pronounced and consistent response. In contrast, *N. elongata*, which was depleted in cannabis smokers in our previous study [4], generally showed weaker molecular and behavioral alterations than the Actinomyces‐treated groups, supporting differences in the patterns associated with the bacterial species examined in this study.

### 3.3 Candidate Microbial Metabolite‐Associated Effects in the Absence of Bacterial Translocation

Despite the behavioral, cellular, and transcriptomic alterations observed following chronic oral exposure to *A. meyeri*, we did not detect any evidence of bacterial translocation from the oral cavity to the brain. No traces of bacterial PGN were detected in the brains of treated mice. Although each of these bacterial detection assays incorporated appropriate positive controls, formal assay‐specific detection limits, spike‐in controls, and inhibition‐control experiments were not performed; such experiments would further strengthen the assessment of low‐abundance bacterial signals and represent a valuable direction for future studies. There is an ongoing debate in the field regarding whether oral bacteria can directly translocate into the brain or whether their systemic effects are mediated primarily through any soluble factor. While some studies have previously reported evidence for oral or gut bacterial presence (P. gingivalis, F. nucleatum) in the brain tissue under conditions of barrier disruption [39, 40], others argue that bacterial metabolites and immune mediators can be translocated to the brain and are sufficient to induce neural alterations without direct microbial invasion [41, 42, 43]. Previous studies have demonstrated that circulating metabolites can access the brain and modulate CNS function [44, 45]. Our findings are consistent with an oral‐brain axis communication model [46] in which soluble products derived from oral dysbiosis with *A. meyeri* influence neural processes without the need for direct bacterial translocation. Notably, *Actinomyces* species are enriched in the saliva of chronic cannabis smokers without any clinical infections in our previous human study [4]. In our current mouse study, chronic oral *Actinomyces* exposure was associated with anxiety‐like behavior and molecular alterations in the absence of detectable bacterial translocation, supporting further investigation of soluble metabolites as potential mediators of oral‐brain communication. If confirmed in humans, prolonged exposure to oral microbiome‐derived metabolites could represent one mechanism by which chronic oral dysbiosis influences neural function.

Integrated comparison of the oral and brain metabolomic datasets revealed compartment‐specific metabolite abundance patterns. Among the metabolites identified, arginine and argininosuccinate were prioritized for further investigation because of their established roles in mitochondrial metabolism and redox homeostasis, together with their abundance patterns across oral and brain compartments. These metabolites were selected as candidate mediators based on the untargeted metabolomics screen and their biological relevance, rather than as definitive markers of oral‐to‐brain metabolite transfer. Both metabolites were also detected in bacterial supernatant, suggesting that Actinomyces species may represent one potential source of metabolites capable of influencing host metabolism. Importantly, the candidate metabolite patterns observed in the brain did not remain significant after correction for multiple comparisons and should therefore be considered exploratory. The relatively small sample size used for brain metabolomics (n = 3 per group) may have limited statistical power to detect metabolite differences after multiple‐testing correction, and these findings require validation in larger independent cohorts.

Arginine metabolism fuels multiple interconnected pathways, including nitric oxide (NO) synthesis, polyamine production, and the urea cycle, all of which directly influence neuronal excitability and survival. Argininosuccinate, as an intermediate in the urea cycle, connects nitrogen disposal with fumarate generation for the TCA cycle. Dysregulation of this axis has been linked to impaired ATP production and accumulation of ROS [47, 48], and elevated levels of urea have been reported in human dementia patients with Lewy bodies [49]. The altered metabolism of arginine [50] and the role of the urea cycle in the brains of patients with AD [51] have also been previously reported. Such perturbations, like imbalances in arginine utilization, shift metabolism toward excessive NO or superoxide production, promoting peroxynitrite formation and redox imbalance [52]. Elevated ROS is well established to impair mitochondrial integrity and has been implicated in both microglial activation and interneuron vulnerability [53].

The other candidate metabolites showed more complex patterns. AICA ribonucleotide, not detected in any bacterial culture supernatants, showed its largest brain abundance in the *A. odontolyticus* group but not in the *A. meyeri*‐treated group. Sedoheptulose‐7‐phosphate, produced by *A. odontolyticus* but not *A. meyeri*, showed only modest changes in the brain despite being detected in both *Actinomyces* species‐treated groups. Neither of these brain metabolite patterns remained significant after multiple‐testing correction. These two metabolites altered mitochondrial ROS generation in Neuro2a cells at the concentration tested, suggesting that they may also contribute to the molecular alterations associated with oral *A. odontolyticus* exposure. Although these findings indicate that AICA ribonucleotide and sedoheptulose‐7‐phosphate may also influence mitochondrial responses, arginine and argininosuccinate were prioritized for subsequent investigation because of their established biological roles in mitochondrial metabolism and redox homeostasis, together with their abundance patterns across the oral and brain compartments. Our findings therefore support further investigation of arginine‐related metabolism, particularly the arginine‐argininosuccinate pathway, as one candidate pathway that may contribute to the mitochondrial remodeling observed in our molecular assays. The metabolite concentrations used in the *Neuro2a* experiments were selected to evaluate dose‐dependent biological activity of candidate metabolites identified through the untargeted metabolomics screen and were not intended to reproduce their in vivo brain concentrations, which remain to be determined by targeted quantitative metabolomics. However, the extent to which these metabolites reach the brain and contribute to the observed molecular and behavioral alterations in vivo remains to be determined. Future studies incorporating plasma and CSF metabolite profiling together with stable isotope‐tracing and bacterial‐labeling approaches will be important to directly establish oral‐to‐brain metabolite transfer.

Taken together, our findings support further investigation of arginine‐related metabolism as a candidate pathway associated with oral *Actinomyces* exposure and the molecular alterations observed in this study. However, targeted metabolite quantification and in vivo tracing studies will be required to determine whether these candidate metabolites directly mediate oral‐to‐brain communication.

In addition, all experimental groups underwent identical antibiotic pretreatment prior to bacterial inoculation to facilitate oral colonization. Therefore, the observed differences between treatment groups are unlikely to be attributable to antibiotic exposure alone. Nevertheless, antibiotic preconditioning together with prolonged oral bacterial administration may have influenced the oral microbial ecosystem beyond the targeted species. Future studies incorporating heat‐killed bacteria, bacterial supernatants, defined microbial metabolites, or non‐antibiotic oral inoculation approaches will help distinguish the relative contributions of live bacteria, changes in the oral microbial ecosystem, and microbial products to the observed phenotypes.

All experiments in this study were conducted in female mice, consistent with our previous work [4]. Sex differences in anxiety‐related behaviors and mitochondrial regulation have been reported, including potential modulation by estrogen‐dependent metabolic pathways [54, 55, 56]. Therefore, it is possible that oral *Actinomyces*‐associated effects may vary between sexes. Notably, similar anxiety‐like behavioral trends have been observed in an independent HIV‐1 Tg26 mouse model following chronic oral *A. meyeri* exposure (unpublished observations from Dr. Sylvia Fitting and colleagues, Behavioral and Integrative Neuroscience, University of North Carolina at Chapel Hill), supporting consistency across experimental systems. Future studies incorporating both sexes will be important to determine the extent to which these effects are generalizable.

## 4 Conclusion

In summary, our findings indicate that oral exposure to Actinomyces, previously identified as enriched in cannabis smokers' oral microbiome [4], is associated with behavioral changes, transcriptional alterations in mitochondrial‐ and GABA‐related pathways, and altered metabolic profiles. These findings occurred in the absence of detectable bacterial translocation, supporting a model in which soluble microbial products may contribute to oral‐brain communication. Although the precise mechanisms underlying this communication remain to be fully defined, several biologically plausible pathways may be involved. One plausible mechanism is that microbial‐derived metabolites enter systemic circulation and, depending on their chemical properties, influence brain function either by crossing the blood‐brain barrier (BBB) through passive diffusion or transporter‐mediated processes, or indirectly through peripheral metabolic or neurovascular signaling. In this context, the cross‐compartment metabolite patterns identified in our untargeted analyses, together with the functional effects of selected candidate metabolites in neuronal‐like cells, support further investigation of microbial metabolites as potential intermediates linking oral microbial changes to central outcomes. However, whether these candidate metabolites reach the brain following oral Actinomyces exposure, their distribution across specific brain regions, their cellular targets, and the mechanisms by which they influence neuronal metabolic and signaling pathways remain to be determined. Future studies integrating plasma and cerebrospinal fluid (CSF) metabolite profiling, targeted quantitative metabolomics, in vivo stable‐isotope‐labeled‐metabolite tracing, BBB transport analysis, and cell‐type‐specific functional assays will be required to define the mechanistic basis of oral‐brain communication and establish the causal contribution of candidate microbial metabolites.

## 5 Materials and Methods

### 5.1 Experimental Animals

C57BL/6J mice were obtained from the Jackson Laboratories and housed in a specified ABSL2 facility with a controlled environment (temperature ranges 68–72 °F and humidity 54%–58%), 12/12 h day‐light cycle with access to food and water ad libitum. The animal study protocol (IACUC‐2019‐00858‐1 and IBC‐2023‐01589) was approved by the institutional committee for animal care and use, and all experiments were conducted according to the guidelines of the National Institutes of Health (NIH) for the care and use of laboratory animals. Before the start of the experiments, mice were kept in the facility for 5 days for acclimatization. Afterward, antibiotics (Amoxicillin‐Vancomycin cocktail 1mg/mL; MUSC pharmacy distribution center) were given to them in drinking water for five consecutive days to reduce the resident oral microbiota, followed by the oral inoculation of the bacteria of interest. Building on our earlier study in female mice [4], we randomly assigned twenty‐four female mice (aged 8–12 weeks) into four groups. They were orally inoculated with 1 × 10^9^ colony‐forming units (CFU) of *Actinomyces meyeri* (ATCC VPI 10648), *Actinomyces odontolyticus (BEI Resource, F0309)*, and *Neisseria elongata (ATCC NCTC 10660)*, with 2% Carboxymethyl cellulose (CMC; Sigma–Aldrich C5678) used as the vehicle control, twice per week for six months. We did not employ germ‐free mice, as their immune immaturity and compromised mucosal defenses markedly increase susceptibility to bacterial colonization in the gut, which would confound our goal of studying oral‐restricted exposure. Using conventionally colonized mice, therefore, allowed us to test oral inoculation effects without altering the broader microbial ecosystem or host immune tone.

Throughout the experiment, body weight changes were monitored weekly, and animal behavior was conducted in a balanced design to cancel out the environmental changes, time, and order effects. Saliva samples were collected after six months via PBS‐soaked cotton swabs to assess sustained oral enrichment of the bacteria of interest by species‐specific qPCR, as described in Section 5.3. At the end of the experiments, mice were anesthetized with 5% isoflurane, and targeted tissues and fluids were harvested carefully. Serum corticosterone levels (Abcam AB108821 corticosterone ELISA) were assessed to monitor their physiological response to sociopsychological stress during the experiments.

### 5.2 Behavioral Tests

#### 5.2.1 Home Cage Locomotor Activity

Home cage activity was monitored for an hour to determine whether the oral bacterial treatment altered spontaneous locomotor activity relative to vehicle‐treated control. Mice were acclimated to the environment 30 min before testing. Individual home cages were placed in an infrared beam‐break activity monitoring chamber (San Diego Instruments, San Diego, California). Spontaneous locomotor activity was quantified as the total number of infrared beam breaks recorded during the testing period using PAS764 software (San Diego Instruments). Because this system uses infrared beam‐break detection rather than video‐based tracking, it quantifies spontaneous ambulatory/locomotor activity but does not generate distance traveled or velocity measurements.

#### 5.2.2 Anxiety‐Like Behavior

Open field test (OFT) and elevated plus maze (EPM) test were performed to determine the anxiety‐like behavior in mice.

The OFT apparatus was divided into three zones, i.e., periphery (outer square), complete center (inner square), and absolute center (innermost square). Each mouse was placed in the absolute center as a starting point, and their locomotor activity was monitored for 5 min under a 120 Lux light source. ANY‐maze software (Stoelting, Chicago, IL) tracked animal position and locomotor scoring.

The EPM test has four arms, i.e., two open arms (without the walls), two closed arms (with the walls), and a small center arena for placing the animal as a starting point. Each mouse was given 5 min under 120 Lux light sources after entering from the center arena, and the time spent in the center, open, and closed arms was determined by video recording and ANYmaze software.

#### 5.2.3 Spatial Learning and Memory

The Y‐maze test and Barnes maze test were performed to determine the spatial working memory, spatial learning, and long‐term reference memory, respectively.

The Y‐maze test has three arms in a Y‐shape, and the spontaneous alternations were determined as a measure of spatial working memory. Mice were placed into the center of the arms, and 5 min were given to them for exploring the maze under a 30 Lux light source. All three arms have a different visual cue to make them distinct from each other. ANYmaze software with a video source was used to calculate spontaneous alternations (the percentage of successive choices where the animals do not repeat an arm entry).

The Barnes maze apparatus is an elevated circular platform with twenty holes. A small, dark escape box is placed under the platform at one of the holes, which is referred to as an escape chamber. Four visual cues are placed on each of the four sides of the Barnes maze with different shapes and colors. This test was conducted under a bright overhead light (at least 450 Lux). All mice underwent a habituation session followed by training trials for five consecutive days, during which they were placed at the center of the maze and allowed to explore until they found the escape hole. If a mouse failed to locate the escape hole within 5 min, it was gently guided to the escape chamber. After training trials, the retention test trial was conducted following a two‐day rest period, similarly to training. The latency to find the escape hole, number of errors, and search strategy were recorded through a video camera, and ANYmaze software was used to assess these measures of spatial learning and memory.

#### 5.2.4 Depression‐Like Behavior

The forced swim test (FST) was performed at the end of the behavioral testing schedule. Mice were placed in the inescapable transparent tank filled with water (controlled temperature between 23°C and 25°C), and their escape‐related mobility behavior was measured for 6 min through video recording. ANYmaze software was used to calculate the immobility time.

All Behavioral testing was performed using a balanced experimental design with automated data acquisition (ANYmaze and PAS software).

### 5.3 Bacterial Quantification in Mouse Saliva

After a week of the last oral inoculation of *A. meyeri*, *A. odontolyticus*, and *N. elongata*, the mice were anesthetized, and oral swab samples were carefully collected from their tongue and gums using PBS‐dipped cotton swabs. Total microbial DNA was extracted from the oral samples using a QIAmp DNA Microbiome kit (Qiagen) according to the manufacturer's protocol. qPCR was performed using a CFX96 Real‐time system (Bio‐Rad). The target sequences were amplified using 10ng of DNA in a 20 µL reaction volume containing Perfecta SYBR Green SuperMix and specific primers (Table S1). Each sample was tested in triplicate. The thermal parameters used for the reaction are as follows: 95°C for 5 min, 40 cycles at 95°C for 15 s, and 60°C for 1 min. To generate a standard curve for each bacterium, genomic DNA was isolated from four serial dilutions of pure bacterial cultures through the same method (starting from 1 × 10^9^ CFU/mL). This genomic DNA was then used in the qPCR assay, and the C_t_ values obtained from the qPCR were employed to generate a standard curve and estimate the CFU‐equivalent abundance of the respective bacteria in mouse oral samples. The serially diluted bacterial DNA served as a positive control to validate assay performance and amplification efficiency. Oral samples from CMC vehicle‐control mice were analyzed in parallel using the same species‐specific primer sets. Prior to behavioral testing, sustained oral enrichment of the inoculated bacterial species was assessed in individual mice by comparing the qPCR‐derived CFU‐equivalent bacterial abundance with the corresponding levels detected in the CMC vehicle‐control group. Only mice meeting the bacterial enrichment criterion and showing bacterial abundance above the corresponding CMC vehicle‐control levels were considered to have sustained oral enrichment and were included in the behavioral and downstream analyses. Animals that did not meet this criterion were excluded prior to behavioral testing.

### 5.4 Detection of Proinflammatory Cytokines

Serum, saliva, brain, and gut samples were obtained from the control and treatment groups at the end of the study. IL‐1β, IL‐6, and TNF‐α concentrations were determined in brain and saliva samples using the U‐plex biomarker multiplex assay (Mesoscale Diagnostics), and cytokine levels in serum and gut were measured using an enzyme‐linked immunosorbent assay (ELISA; Abcam). The respective detection procedures followed the manufacturer's instructions.

### 5.5 Brain Sample Embedding and Sectioning

Mouse brains were sagittally cut into two halves, and one half was used to embed in formalin‐fixed paraffin‐embedded blocks using a standard protocol. Tissue sections were cut (5 µm) and mounted on charged slides. RNA in situ hybridization and immunohistochemical staining were performed as below:

#### 5.5.1 RNA in Situ Hybridization

The RNAScope 2.5 High Definition (HD) chromogenic duplex and multiplex fluorescent assays (ACDBio) were performed to visualize target RNA in the brain sections of control and treatment mouse groups. FFPE slides were pre‐treated (including target retrieval step) according to the manufacturer's protocol and followed by subsequent multiple steps of probe hybridization and signal development through HRP channel binding. The experiment consisted of hybridizing multiple probes, i.e., Iba1 (Mm‐1089911), Tmem119 (Mm‐472901), and unique 16s rRNA sequence probes for *A. meyeri* (B‐1315481), *A. odontolyticus* (B‐1328481), and *N. elongata* (B‐1328471), along with manufacturer‐provided positive (321811) and negative (321831) RTU control probes set to verify assay performance and specificity. TSA vivid fluorophores were used to label different channels in the fluorescent assay, i.e., TSA plus fluorescein (green) with Iba1, TSA plus cyanine 3 (orange) with Tmem119, and TSA plus cyanine 5 (far red) with the respective 16s rRNA bacterial probe in a 1:1500 dilution. DAPI was used as a counterstain for nuclei. For the chromogenic assay, to demonstrate whether any brain region is specific to microglial activation, the Iba1 probe (1089911‐C1) was used, and HRP‐enzyme‐based detection was performed. Hematoxylin I was used as a counterstain, and stained sections were carefully mounted on the slides for visualization. Confocal microscopy was done using LSM Meta 550 and Leica Stellaris 8 for multiplex assay, while Leica Thunder's bright‐field microscopy was used for the chromogenic assay. Quantification of images was performed by an investigator blinded to the treatment groups.

#### 5.5.2 Immunohistochemistry

Brain sections underwent deparaffinization and rehydration in different concentrations of xylene and ethanol, as previously described [4]. After the heat‐induced antigen retrieval step, the sections were washed with TBST and blocked with 10% normal serum (1% BSA in TBS) for an hour. Different sections were stained with Rabbit monoclonal to Amyloid precursor protein (ab32136) and rabbit monoclonal antibody to Iba1 (ab178846) in a 1:500 dilution overnight at 4°C. After washing, goat anti‐Rabbit IgG H&L (Alexa Fluor 488) was used (ab150077) in a 1:1000 dilution as a secondary antibody for an hour at room temperature. After the final wash steps, Vectamount mounting medium with DAPI (Vector Laboratories) was used, and the slides were cover‐slipped. The Zeiss LSM 510 meta confocal microscope was used to visualize the signal. ZEN Software was used for image acquisition, and ImageJ was used for quantification of the signal by an investigator blinded to the treatment groups.

### 5.6 Single‐Cell RNA Sequencing

Whole half‐brains from three mice within each experimental group (Vehicle, *A. meyeri*, *A. odontolyticus*, and *N. elongata*) were pooled prior to nuclei isolation, generating one integrated sample per experimental condition. Mouse brain samples from control and treatment groups were snap‐frozen and submitted for single‐cell RNA sequencing to Singulomics Corporation (Bronx, NY). The nuclei were isolated from the frozen mouse brain tissue and constructed 3’ single‐cell gene expression libraries (NEXT‐GEN v3.1) using the 10X Genomics Chromium‐X system. The libraries were sequenced with ∼200 million PE150 reads per sample on Illumina NovaSeq X Plus. The sequencing reads were analyzed with the mouse reference genome (GRCm39‐2024‐A). These reads include the addition of organism‐specific transgene reference sequences for detecting *A. meyeri*, *A. odontolyticus*, and *N. elongata* using Cell Ranger v8.0. This approach enabled the detection of reads corresponding to these bacterial species, if present, within the single‐cell sequencing dataset. Introns were included in the analysis.

The analysis workflow initiates with data processing through Cell Ranger, followed by rigorous quality control and additional data refinement. For the sample analysis, the Seurat workflow was employed, which includes normalization using the sctransform v2 pipeline. Subsequently, Louvain clustering was performed, enabling the identification of distinct cell populations within the sample. To further validate the cell types, the Azimuth application and Seurat's anchor‐based label transfer method were employed [57, 58]. Besides that, cell types were identified using a reference dataset obtained from a similar tissue, specifically the mouse motor cortex. This reference dataset was constructed using data and annotations provided by the Allen Brain Institute and encompasses 159 378 nuclei integrated across 12 individual mice [59].

After sample integration with the Harmony algorithm, we obtained the UMAP plots for each condition/treatment. Because each condition consisted of a single pooled biological sample, animal‐level pseudobulk or mixed‐effects analysis was not possible. Therefore, the scRNA‐seq analyses were used as an exploratory transcriptomic screen to identify candidate pathways and genes for subsequent validation. Genes were ranked using the Seurat FindMarkers() function with the Wilcoxon Rank Sum test at the cell level. Because the resulting p‐values and adjusted p‐values reflect variation among cells/nuclei within the pooled samples rather than variation among individual animals, they were not interpreted as animal‐level statistical inference. Genes were prioritized for exploratory downstream analysis based on an absolute log2 fold‐change > 0.25 together with cell‐level ranking statistics. Gene Set Enrichment Analysis (GSEA) was performed to identify candidate biological pathways. The DAVID bioinformatics tool was used for functional annotation of KEGG and Reactome pathways, including the visualization of prioritized genes on pathway maps.

### 5.7 Estimation of Brain GABA Levels

GABA levels in mouse whole brain lysates were quantified using a commercially available ELISA kit (Biomatik, EKF58282). Protein concentrations were first determined using the BCA assay, and sample dilutions were optimized to ensure accurate detection within the assay's dynamic range. Following ELISA quantification, raw GABA concentrations (pg/mL) were adjusted according to the dilution factor and normalized to total protein content. Final GABA levels were expressed as micromolar (µm) per 100 µg of total protein, allowing for direct comparison with existing literature [60].

### 5.8 Detection of Peptidoglycan in the Brain

Bacterial peptidoglycan in mouse brain lysates was determined using a commercially available Silkworm Larvae Plasma (SLP)‐HS Single Reagent Set II (FUJIFILM Wako Chemicals). The assay principle is that the hemolymph of the silkworm Bombyx mori contains a self‐defense mechanism known as the “prophenoloxidase (proPO) activating system” or “proPO cascade”. Upon invasion by a microbe, the system participates in melanin formation, as observed in the body fluids of insects, to protect them from the invader's attack. The system is activated by peptidoglycan (PGN) from bacteria, and consequently, proPO in the system is activated. The system is thought to be a cascade reaction involving the activation of multiple zymogens of proteinases. The SLP reagent is a lyophilized product containing factors of the proPO cascade. It is prepared from the body fluid of silkworm larvae and processed without melanin formation. PGN activates the SLP reagent, then L‐3,4‐dihydroxyphenylalanine (DOPA) is oxidized, and melanin is formed. Since PGN is found not only in gram‐positive but also gram‐negative bacteria, the SLP reagent responds to a wide range of bacteria, regardless of their Gram‐staining classification. Therefore, this kit is used to detect the presence of PGN by measuring the melanin pigment formation of the SLP reagent as an indicator. As a positive control, pure cultures of lysed bacteria (*A. meyeri, A. odontolyticus, and N. elongata*) were used to verify assay performance.

### 5.9 Western Blotting

Mouse whole brain lysates were prepared using RIPA lysis buffer (ThermoFisher 89900) supplemented with a protease/phosphatase inhibitor cocktail. Protein estimation of the samples was performed using the BCA method. An equal amount of protein (25 µg) was resolved by SDS‐PAGE and transferred to PVDF membranes (Millipore). Membranes were blocked with 5% skim milk in TBS‐T, incubated with primary antibodies overnight at 4 °C, and then incubated with HRP‐conjugated secondary antibodies (Cell Signaling Technology) the next day. Bands were visualized using ECL (Thermo) and quantified with ImageJ. Primary antibodies are Cox IV mouse mAb # 1967 (4D11‐B3‐E8, CST), ANT1 polyclonal Ab (PAS‐109394, Thermo), GAD1 (#5305S, CST), GAD2 (D5G2) XP Rabbit mAb (#5843S, CST), and GAT1 Rabbit mAb (373425, CST). Secondary antibodies are anti‐rabbit (Cat #7074S, CST) and anti‐mouse (Cat #7076S, CST).

### 5.10 Screening of Metabolites in the Brain, Saliva, and Bacterial Supernatants

Mice saliva samples were collected as described in detail previously [4]. Brain samples were harvested, and homogenization of frozen tissues was performed under liquid nitrogen. These samples were sent to Metabolon Inc. (Durham, NC) for the metabolomic study. Whole‐brain samples from three mice per experimental group and saliva samples from six mice per group were submitted for untargeted metabolomic profiling. Samples were prepared using the automated MicroLab STAR system. Proteins in the sample were precipitated (with the aid of a small molecule matrix) using methanol under vigorous shaking for 2 min, followed by centrifugation. The resulting extracts were subjected to different chromatographic and spectrometric pipelines as previously described by Ford and colleagues, 2020 [61]. Briefly, the untargeted metabolomics analyses were performed using ultrahigh‐performance liquid chromatography/tandem mass spectrometry (UPLC‐MS/MS) and gas chromatography‐mass spectrometry (GC‐MS), following their standard procedures. Reference libraries were used to identify different metabolites with their raw peak values. Metabolite abundance was quantified using normalized relative peak areas (area under the curve). For both oral and brain metabolomic datasets, metabolites were analyzed using ordinary one‐way ANOVA across the four experimental groups, followed by planned treatment‐versus‐vehicle contrasts using the pooled ANOVA residual variance. Benjamini‐Hochberg FDR correction was applied separately across the full set of metabolites tested within each treatment‐versus‐vehicle contrast.

*Actinomyces meyeri* and *A. odontolyticus* were cultured in the Actinomyces‐specific broth (HIMEDIA M233) for 48 h. Bacterial cells were pelleted down, and supernatants were filtered out (0.22 µm) for the untargeted metabolomics study executed by Creative Proteomics Inc. through UPLC‐MS. Separation was performed by ACQUITY UPLC (Waters) combined with Q‐Exactive MS (Thermo) and screened with ESI positive and negative modes, provided with both Hilic and C18 columns.

### 5.11 In Vitro Assays of Mouse Neuroblastoma Cell Lines

Neuro2a (ATCC CCL‐131) cell line was maintained in DMEM medium (Cat # 11965‐092) with 10% FBS and 5% PSN antibiotic solution (Cat # 15640‐055) and grown until the cells looked healthy and confluent in cell culture‐treated plates (Cat # 240304; 100 × 15 mm) at 37°C and 5% CO2 in a humid atmosphere.

#### 5.11.1 DCFDA‐ROS Assay

The cellular reactive oxygen species (ROS) levels were determined using the DCFDA/H2DCFDA ROS assay kit (Abcam ab113851). TBHP 200 µm and H2O2 400 µm were used as positive controls. Briefly, Neuro2a cells were plated (seeding density 25 000 cells per well) in an FBS‐supplemented DMEM without phenol red medium (Cat # 21063‐029) onto a 96‐well black plate (Cat # 3603 Corning Costar). After 24 h, cells were washed with 1x wash buffer (provided in the kit) and incubated with 10µM DCFDA for 45 min at 37°C (in the dark). The DCFDA solution was removed carefully, and different treatments of interest (metabolites in various concentrations) in the same FBS‐supplemented, phenol red‐free DMEM medium were added to their respective wells. ROS production was determined immediately by measuring the formation of fluorescent dichlorofluorescein (DCF), using a spectrophotometer at 458/535 excitation/emission wavelengths for 6 h (every 2 h). DCFDA assays were performed in three independent experiments conducted on separate plates, with each treatment condition analyzed in three technical replicate wells per experiment. Fluorescence intensity values were normalized to cell number based on equal seeding density across wells and are presented as relative fluorescence units (RFU).

#### 5.11.2 MitoSox ROS Assay

The mitochondrial superoxide (MitoSox; refers to the reactive oxygen species (ROS) generated specifically in mitochondria) levels were determined using the Mitochondrial Superoxide Fluorometric Assay Kit (Abcam ab219943). Antimycin A 50µM was used as a positive control. The procedure for seeding the N2a cells was essentially the same as in the DCFDA assay; however, a 5 × 10^5 seeding density per well was used. After 24 h, the old media was gently replaced with fresh complete media (without phenol red), containing treatments (different metabolites) or antimycin A, and incubated for 30 min at 37°C in the dark for superoxide production. Afterward, 100 uL/well diluted MitoROS staining solution was added, and the cells were further incubated at 37°C for 30 min, protected from light. After incubation. endpoint fluorescence intensity was measured at Ex/Em = 540/590 nm. MitoSOX assays were performed in three independent experiments conducted on separate plates, with each treatment condition analyzed in six technical replicate wells per experiment. Fluorescence intensity values were normalized to cell number based on equal seeding density across wells and are presented as relative fluorescence units (RFU). Mitochondrial mass was not independently quantified in this assay.

#### 5.11.3 Mitochondrial Function Assays

Oxygen consumption rates (OCRs) were measured using an Agilent Seahorse XFe96‐Pro analyzer according to the Agilent Mito stress test protocol. Neuro2a cells were seeded onto the Seahorse 96‐well plate at a density of 40 000 cells per well for 2 h after treatment with different concentrations of the metabolite of interest and then incubated for 22 h. One hour before the assay, plated cells were incubated at 37°C in the absence of carbon dioxide. Cells were sequentially treated with mitochondrial poisons, i.e., Oligomycin (1.5 µm), Carbonyl cyanide‐4 (trifluoromethoxy) phenylhydrazone (FCCP) (1.5 µm), Rotenone, and Antimycin (0.5 µm), while measuring the OCR through the Seahorse instrument. (a) Basal respiration was determined by subtracting the non‐mitochondrial OCR (after rotenone and antimycin A treatment) from the basal OCR (before any mitochondrial poison treatment); (b) ATP synthesis by subtracting the OCR after oligomycin from the baseline OCR; (c) Proton Leak by subtracting non‐mitochondrial OCR from the OCR after oligomycin; (d) Maximal respiration by subtracting non‐mitochondrial OCR from the OCR after FCCP; (e) Spare respiratory capacity by subtracting basal respiration from the maximal respiration. Seahorse assays were performed in three independent experiments conducted on separate plates, with each treatment condition analyzed in 10 technical replicate wells per experiment. Phase‐contrast microscopy was performed on the plate to assess the cell confluency and confirm the equal seeding density through CellProfiler software for normalization of OCRs.

### 5.12 Statistical Analysis

Statistical analysis was performed using GraphPad Prism version 11, and data are presented as mean ± S.E.M. unless otherwise specified in the respective Figure legend. Comparisons between the groups were performed using two‐tailed unpaired Student's t‐test, one‐way analysis of variance (ANOVA), two‐way ANOVA, mixed‐effects model, or repeated measures design with Tukey's multiple‐comparison tests where applicable. For the OFT, absolute‐center time and periphery time were additionally analyzed using analysis of covariance (ANCOVA), with total distance traveled during the OFT included as a covariate to account for locomotor activity. Homogeneity of regression slopes was assessed before comparing adjusted group effects. The statistical analysis and multiple‐testing correction applied to the untargeted metabolomic datasets are described in Section 5.10. The scRNA‐seq data were analyzed using the bioinformatic procedures described in Section 5.6 and were treated as exploratory; cell‐level statistical values were used for gene ranking and prioritization rather than animal‐level statistical inference. For other analyses, significance was defined as *p* < 0.05 unless otherwise specified. Image acquisition and quantification (ImageJ/Fiji) were conducted in a blinded manner, with identical acquisition settings and standardized analysis parameters applied across all groups to ensure unbiased measurements. N2a cell‐based experiments were performed in three independent experiments (*n* = 3). For DCFDA, MitoSOX, and Seahorse assays, each treatment condition was analyzed in three, six, and ten technical replicate wells per independent experiment, respectively. Information on the sample size, number of biological replicates, and the statistical test used for each experiment is included in the figure legends.

## Author Contributions

**Tabinda Salman**: methodology, investigation, visualization, formal analysis, validation, Writing – original draft, Writing – review and editing, data curation. **Zhenwu Luo**: investigation. **Douglas Johnson**: investigation. **Arshad A. Noorani**: methodology. **Zhuang Wan**: formal analysis, visualization, software. **Bogdan Bordieanu**: investigation. **Zhi‐wei Ye**: investigation, writing – review and editing. **Rachel D. Penrod**: methodology, writing – review and editing. **Hongxu Xian**: methodology, writing – review and editing. **Sylvia Fitting**: conceptualization, supervision, funding acquisition, writing – review and editing. **Peter W. Kalivas**: writing – review and editing. **Wei Jiang**: conceptualization, supervision, funding acquisition, writing – review and editing.

## Funding

National Institute on Drug Abuse grant R01DA059854 (WJ), National Institute on Drug Abuse grant R01DA059538 (WJ), National Institute on Drug Abuse grant R01DA055523 (SF, WJ), 2026 NARSAD Distinguished Investigator Grant from the Brain & Behavior Research Foundation (34152, WJ), National Institute on Drug Abuse grant K24DA065777 (WJ).

## Conflicts of Interest

The authors declare no conflicts of interest.

## Supporting information




**Supporting File 1**: advs78144‐sup‐0001‐S5.zip.

**Supporting File 2**: advs78144‐sup‐0002‐S6.zip.

**Supporting File 3**: advs78144‐sup‐0003‐SuppMat.docx.

## Data Availability

The data that support the findings of this study are available in the supplementary material of this article. The raw files regarding the behavioral assessments and untargeted metabolomics screening in the mouse brain have been deposited in Figshare and are publicly available at: https://doi.org/10.6084/m9.figshare.30676025. The scRNA‐seq raw and processed data files are available through GEO accession no. GSE347327.
