# Supplementary material for: Oral Actinomyces Species Exposure is Associated with Anxiety‐Like Behavior, Mitochondrial Remodeling, and Altered GABAergic Pathways
Source: Adv Sci (Weinh). 2026 Oct 14:e78144. Online ahead of print. doi: 10.1002/advs.78144 (PMC13668766; doi:10.1002/advs.78144)
Supplement: Supplementary file 1 [file ADVS-9999-e78144-s002.docx]

**Supplemental Information**

**Title**

Oral Actinomyces species exposure is associated with anxiety-like behavior, mitochondrial remodeling, and altered GABAergic pathways

**Authors**

*Tabinda Salman^1^, Zhenwu Luo^1^, Douglas Johnson^1^, Arshad A. Noorani^1^, Zhuang Wan^1,5^, Bogdan Bordieanu^2^, Zhi-wei Ye^1^, Rachel D. Penrod^2^, Hongxu Xian^3^, Sylvia Fitting^4^, Peter W. Kalivas^2^, Wei Jiang^1,5^****^*^***

**Supporting Information includes**

Supplementary Text

Figures S1 to S6

Table S1

**Other Supplementary Material for this manuscript includes the following separately:**

Raw Data Files for Figures 3 and S4.

Raw Data Files for Figures 5 and S5.

**Supplementary Figure S1: Quantification of bacterial DNA in saliva (oral swabs) and fecal (stool) samples after oral inoculations.**

Oral swab samples were collected from mice after chronic oral inoculation of the microbiome, while feces were collected from the intestines at the time of mouse sacrifice (fresh catch). The quantification of *A. meyeri, A. odontolyticus*, and *N. elongata-*specific DNA was evaluated using qPCR with their specific primers. To generate a standard curve for each bacterium, genomic DNA was isolated from four serial dilutions of pure bacterial culture through the same DNA extraction method (starting from 1 x 10^9^ CFU/mL). The resulting Ct values were used to generate standard curves and estimate the CFU-equivalent abundance of the respective bacteria in mouse oral samples. Data are presented as mean ± SEM (n = 6 biological samples per group). Statistical comparisons were performed between the respective bacterial treatment and vehicle control using two-tailed Mann-Whitney U tests. **p < 0.001.

**D.**

**C.**

**B.**

**A.**

**Supplementary Figure S2. No prominent changes in mouse behaviors related to cognitive function, depression-like behavior, or serum corticosterone levels.**

(A) Measurements obtained before initiation of the behavioral test battery. Home-cage locomotor activity was increased in all bacterial treatment groups compared with vehicle control (F*_(3,20)_* = 10.12, p = 0.0003). All mice gained body weight throughout the six-month oral treatment period with *A. meyeri*, *A. odontolyticus*, or *N. elongata*. No significant differences in serum corticosterone levels were observed among the groups. (B) Y-maze testing showed no significant differences in spontaneous alternation among the bacterial treatment groups compared with vehicle control. (C) Multiple parameters were assessed in the Barnes maze during the recall trial following five days of training. No significant group differences were observed in the parameters examined. (D) The forced swim test showed a trend toward increased immobility time in the *A. meyeri* group, but this did not reach statistical significance. Data are presented as mean ± SEM (n = 6 biological samples per group). **p < 0.01, ***p < 0.001; one-way ANOVA followed by Tukey’s multiple-comparisons test.

**Supplementary Figure S3: Chronic oral inoculation of *A. meyeri* is associated with altered microglial markers without evidence of macrophage infiltration in the mouse brain.**

(A) RNA-based in situ hybridization (RNAScope). Representative images of 5 μm FFPE sagittal mouse brain sections are shown. Sections were stained for IBA1 (activated microglia/macrophages; FITC) and TMEM119 (resident microglial marker; Cy3), with DAPI as a nuclear counterstain. Scale bar = 100µm (brain cortex). (B) Quantification of mean fluorescent intensities (MFI) using ImageJ/Fiji showed increased IBA1 and TMEM119 signals in the *A. meyeri-inoculated* group compared with control groups. No prominent population of IBA1+ cells lacking TMEM119+ co-localization was detected, providing no evidence of systemic macrophage infiltration into the brain. (C) Immunohistochemical staining of IBA1 in representative images of 5-μm FFPE sagittal mouse brain sections. Sections were immuno-stained with IBA1 antibody and DAPI as a nuclear counterstain. Scale bar = 50µm. (D) MFI analysis of IBA1 immunohistochemical staining using ImageJ/FIJI showed increased IBA1 protein signal in the *A. meyeri*-treated group compared with vehicle controls. Brain tissue from three mice per group was analyzed (n = 3 biological replicates per group). Three sections from each mouse were examined, with multiple images acquired from each section and quantified using ImageJ/Fiji. Data are presented as mean ± SEM. **p < 0.01, ***p < 0.001, ****p < 0.0001; one-way ANOVA followed by Tukey’s multiple-comparisons test.

**Supplementary Figure S4: IBA1-positive signal across multiple brain regions following chronic oral *A. meyeri* inoculation.**

RNA-based in situ hybridization (RNAScope). Representative 5-µm FFPE sagittal brain sections from mice treated with vehicle or *A. meyeri* for six months are shown. Images were acquired using a Leica Thunder (brightfield) microscope at 5x, 10x, 20x, and 40x magnification to visualize IBA1-positive signal (RNAScope probe # 1089911-C1) across multiple brain regions. Chromogenic signals were converted to grayscale using ImageJ/Fiji for better visualization. HRP-based detection was performed, and hematoxylin I was used as a counterstain. Representative areas from sagittal brain sections of vehicle- and *A. meyeri*-treated mice are shown at increasing magnification from 5× to 40× to illustrate the distribution of IBA1-positive signal across different brain regions. Brain tissue from three mice per group was examined (n = 3 biological replicates per group), with three sections analyzed from each mouse.

**Supplementary Figure S5: Cell-type composition and exploratory scRNA-seq comparisons of *A. odontolyticus* and *N. elongata* relative to *A. meyeri.***

(A) Cell numbers obtained from each pooled sample and corresponding UMAP plots annotated by cell types. Cell populations are colored by treatment conditions. (B) UMAP plots of the integrated dataset split by individual treatment conditions. Twenty-two distinct cell types were identified and are shown in different colors. (C) Exploratory KEGG pathway-enrichment analysis based on prioritized genes showing increased (pink) or decreased (blue) relative expression across the indicated treatment comparisons. Circle size represents gene count, and the color scale represents −log10(p-value) from the pathway-enrichment analysis. (D) Exploratory Reactome/biological- process enrichment analysis using the DAVID bioinformatics functional annotation tool based on prioritized genes showing increased or decreased relative expression. Circle size represents gene count, the color scale represents −log10(p-value), and enrichment scores are derived from the geometric mean of the annotation p-values. (E) Heatmaps showing prioritized genes with increased or decreased relative expression across different cell types based on average log2 fold-change in each integrated treatment comparison. Whole half-brains from three mice per group were pooled to generate one sample per treatment condition; therefore, these scRNA-seq comparisons are exploratory and do not represent animal-level statistical inference. Pathway-level p-values were used as exploratory annotation and prioritization metrics and were not interpreted as animal-level statistical significance.

**Supplementary Figure S6: No detectable *A. odontolyticus* or *N. elongata* 16S rRNA signal in the mouse brain, and detection of selected candidate metabolites in bacterial culture supernatants.**

(A) Representative images of 5-µm FFPE sagittal mouse brain sections analyzed by RNAScope in situ hybridization. No detectable *A. odontolyticus* or *N. elongata* 16S rRNA signal was observed in the brain sections under the conditions examined (Cy5; yellow), with DAPI shown in blue. A UBC (Ubiquitin C) positive-control probe targeting a housekeeping gene was used as an assay control in the Cy5 channel; representative positive-control images are shown with the *A. meyeri* brain sections in Figure 4. Images were acquired at 40× using a Zeiss LSM 510 Meta confocal microscope (1024 × 1024 pixels; n = 4 biological samples per group). (B) Of the 14 candidate metabolites detected in both saliva and brain, 10 were also detected in bacterial culture supernatants. Metabolite abundance was normalized to the corresponding basal growth medium and is presented as fold change. Argininosuccinate was detected in *A. meyeri* culture supernatants by UPLC-MS under the in vitro conditions examined. Bacterial culture supernatants were analyzed from three independent samples per condition (n = 3). Individual graphs show selected metabolites shared between mouse oral swabs and brain samples.

**Supplementary Table 1: The oligos used for qPCRs**

**Other Supplementary Materials:**

**Data Files for Fig. 3 and Fig. S4.**

List of prioritized genes showing altered relative expression across the group-wise comparisons in each cell type identified in the scRNA-seq analysis.

**Data Files for Fig. 5 and Fig. S5.**

List of metabolites along with their raw peak values in mouse saliva, brain, and pure bacterial supernatants.
